# The Location of Magnetic Reconnection at Earth’s Magnetopause

**DOI:** 10.1007/s11214-021-00817-8

**Published:** 2021-03-29

**Authors:** K. J. Trattner, S. M. Petrinec, S. A. Fuselier

**Affiliations:** 1 LASP, University of Colorado, Boulder, CO USA; 2 Lockheed Martin ATC, Palo Alto, CA USA; 3 Southwest Research Institute, San Antonio, TX USA; 4 University of Texas at San Antonio, San Antonio, TX USA

**Keywords:** Magnetic reconnection, Reconnection location, Plasma entry into magnetosphere, Precipitating ions, Boundary layer

## Abstract

One of the major questions about magnetic reconnection is how specific solar wind and interplanetary magnetic field conditions influence where reconnection occurs at the Earth’s magnetopause. There are two reconnection scenarios discussed in the literature: a) anti-parallel reconnection and b) component reconnection. Early spacecraft observations were limited to the detection of accelerated ion beams in the magnetopause boundary layer to determine the general direction of the reconnection X-line location with respect to the spacecraft. An improved view of the reconnection location at the magnetopause evolved from ionospheric emissions observed by polar-orbiting imagers. These observations and the observations of accelerated ion beams revealed that both scenarios occur at the magnetopause. Improved methodology using the time-of-flight effect of precipitating ions in the cusp regions and the cutoff velocity of the precipitating and mirroring ion populations was used to pinpoint magnetopause reconnection locations for a wide range of solar wind conditions. The results from these methodologies have been used to construct an empirical reconnection X-line model known as the Maximum Magnetic Shear model. Since this model’s inception, several tests have confirmed its validity and have resulted in modifications to the model for certain solar wind conditions. This review article summarizes the observational evidence for the location of magnetic reconnection at the Earth’s magnetopause, emphasizing the properties and efficacy of the Maximum Magnetic Shear Model.

## Introduction

**Fig. 1:**
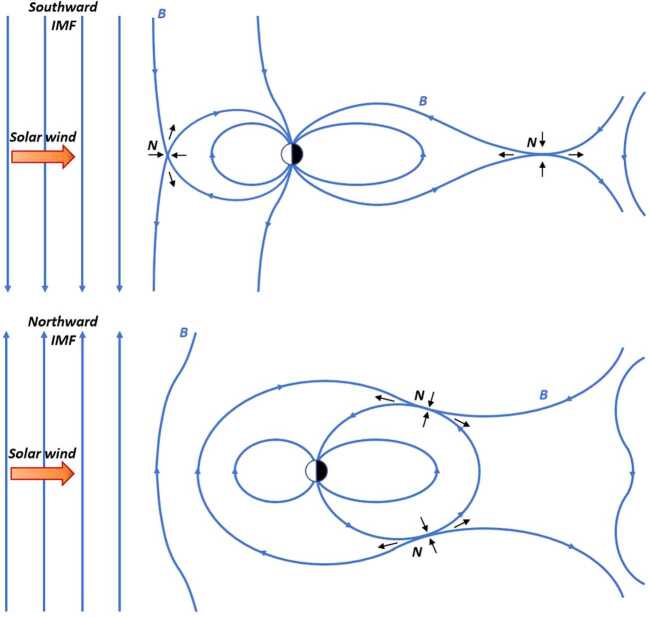
Early 2D representations of reconnection between the magnetic fields (blue traces) of the solar wind and the Earth’s magnetosphere, as first described by Dungey (1961, 1963). Top: A pure southward IMF condition, showing magnetic reconnection occurring at null points (N) in the subsolar region and within the magnetotail, with associated magnetic field motion and plasma inflow and outflow (represented by black arrows). Bottom: An instance of pure northward IMF, showing magnetic reconnection at high latitudes just downstream of the Earth. Adapted from Russell (2000)

Collisionless magnetic reconnection occurs between the shocked solar wind plasma in the magnetosheath and the terrestrial magnetospheric plasma in the magnetosphere. This reconnection occurs at the magnetopause boundary between these two plasma regimes. The initial schematic portrayal of this process was presented in 2D (Dungey 1961, 1963), with reconnection of oppositely oriented solar wind and geomagnetic field lines. Reconnection was thought to occur in a small region where the plasma becomes demagnetized, creating “open” magnetic field lines that extend from the Earth’s ionosphere into the solar wind. As shown in the top portion of Fig. 1 (adapted from Dungey 1961), this initial concept placed the reconnection point at the magnetopause standoff location when the southward-directed interplanetary magnetic field (IMF) was opposite to the northward-directed intrinsic magnetic field of the Earth. The standoff location represents the “first point of contact” of the shocked solar wind flow and its embedded magnetic field against the Earth’s compressed magnetic field at the magnetopause boundary.

For pure northward IMF ($+\text{Z}$-direction) conditions, magnetic reconnection was proposed to occur just downstream of the Earth at high latitudes as the solar wind IMF drapes the high-altitude extension of the Earth’s intrinsic magnetic field. This reconnection geometry is depicted in the bottom portion of Fig. 1 (adapted from Dungey 1963).

However, it was realized that conditions favorable for the occurrence of steady magnetic reconnection at the magnetopause extend beyond singular locations at the magnetopause, and require the consideration of the full 3D configuration. In particular, for a pure southward IMF and for zero-degree tilt angle of the Earth’s dipole magnetic field, magnetic reconnection could take place all along the dayside geomagnetic equator where the magnetic fields internal and external to the magnetopause are anti-parallel to one another. This geometry is depicted as an extended reconnection line (also often described as a continuous “X-line”).

**Fig. 2:**
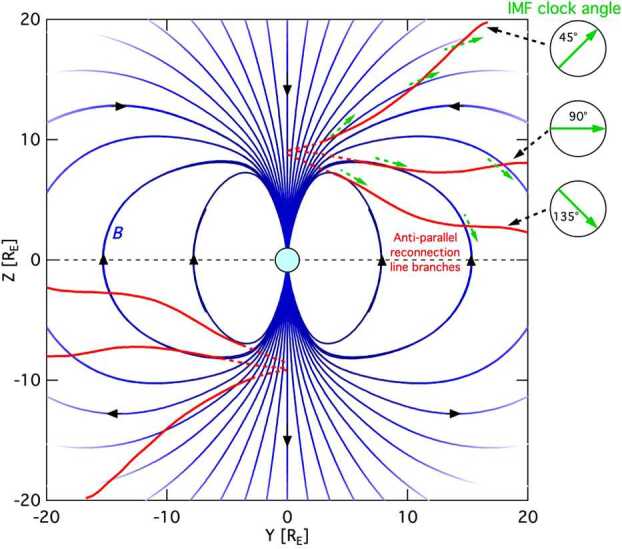
A view of the magnetosphere as viewed from the Sun. For various IMF clock angles (green), the draped solar wind magnetic field about the magnetopause results in high-latitude regions where anti-parallel reconnection may occur (red). Dashed segments correspond to the magnetospheric cusp funnel regions. This is a representation of an equinox interval, with a magnetospheric magnetic dipole moment angle of zero degrees. Adapted from Crooker (1979), and Luhmann et al. (1984)

The condition of pure southward (or northward) IMF is extremely rare in reality; the general IMF vector normally includes nonzero components in directions orthogonal to the Z-direction, with the Geocentric Solar Magnetospheric (GSM) system the most practical for organizing magnetic field orientations. Investigations of the effect of a nonzero IMF B_Y_-GSM component during southward IMF on the location of anti-parallel magnetic reconnection at the magnetopause were conducted by Crooker (1979) and Luhmann et al. (1984) (again, for a zero-degree tilt angle of Earth’s dipole magnetic field). In these model scenarios, magnetic reconnection does not occur at the standoff location near the subsolar magnetopause (since the magnetic fields in the two regions are not anti-parallel to one another); but rather along two extended branches. As depicted in Fig. 2, each branch represents a contiguous reconnection line (shown in red), beginning near the high-latitude cusp near local noon and extending out along the magnetopause flanks. Each line traces out the loci of points where anti-parallel reconnection can occur. For increasingly northward IMF conditions, these reconnection branches extend to higher latitudes and further behind (tailward of) the Earth (also shown in Fig. 2). In general, the model’s two reconnection line branches are mirror images about both the local noon/midnight meridian and the geomagnetic equator.

**Fig. 3:**
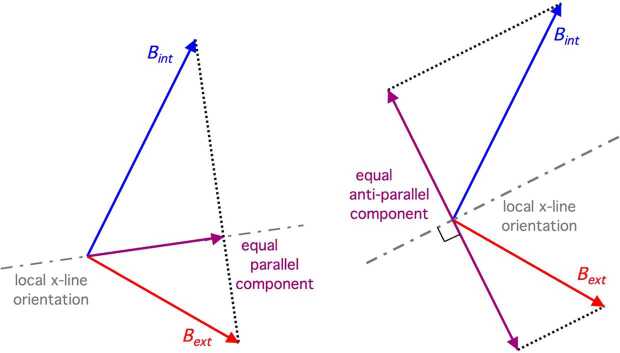
Early models of the component reconnection tilted X-line orientation. Left: X-line orientation aligned with the equal and parallel components of the internal and external magnetic fields (Sonnerup 1970; Gonzalez and Mozer 1974; Hill 1975). Anti-parallel components exist but are generally of unequal magnitude. Right: X-line orientation aligned perpendicular to the equal and opposite components of the internal and external magnetic fields (Moore et al. 2002)

An alternative concept to the above scenario has also been suggested and investigated. The central tenet of this concept is that the magnetic fields internal and external to the magnetopause need not be anti-parallel to one another in order to initiate collisionless magnetic reconnection. Instead, there merely needs to exist some significant component of the merging magnetic fields that are anti-parallel to one another. This description maintains that magnetic reconnection under southward IMF conditions would still occur at low latitudes even with a nonzero IMF B_Y_-GSM component. Reconnection would take place where the solar wind first makes contact with the magnetopause, and extend along a single contiguous reconnection line emanating from the standoff location towards the flanks at low latitudes. This orientation of the reconnection line is related to the IMF direction, and is often called a “component reconnection tilted X-line” or simply a “tilted X-line” model. In one variation of the scenario, the tilted X-line is constrained to align along the direction where a component of the magnetic fields on the two sides of the magnetopause are parallel to one another and of equal strength. This scenario also requires that the remaining component of the magnetic fields tangent to the magnetopause are anti-parallel – but not necessarily of equal strength (Sonnerup 1974; Gonzalez and Mozer 1974; Hill 1975). This is depicted in the left portion of Fig. 3. This restriction of parallel components was relaxed in another variation to the tilted X-line scenario by Cowley (1976) and Cowley and Owen (1989), with the only constraint being that some component of the two magnetic fields are anti-parallel. Moore et al. (2002) imposed a different tight constraint; that one component of the magnetic fields on the two sides of the magnetopause must be anti-parallel to one another and of equal strength (right portion of Fig. 3).

Tilted X-lines were typically described as straight lines as viewed from the Sun. This description was challenged by Moore et al. (2002). They used a more realistic draping of the shocked IMF around the magnetopause along with a more realistic magnetospheric magnetic field as described by the Tsyganenko (T96) semi-empirical model (Tsyganenko 1995). Pressure balance arguments were also used to locally set $|\text{B}_{\text{Magnetosheath}}| = |\text{B}_{\text{Boundary}\_\text{Layer}}|$. The resulting reconnection line traces for zero-dipole tilt but varying IMF B_Y_ and B_Z_ conditions (also described as the IMF clock angle: $\tan^{-1}(\text{B}_{\text{Y}}/\text{B}_{\text{Z}})$) are curved while passing through the magnetopause standoff location.

For the component reconnection tilted X-line scenarios, it is expected that there is an IMF clock angle limit beyond which there is a sufficient northward component that steady reconnection ceases to occur equatorward of the cusps, and instead preferentially occurs along the anti-parallel regions poleward (tailward) of the cusps.

**Fig. 4:**
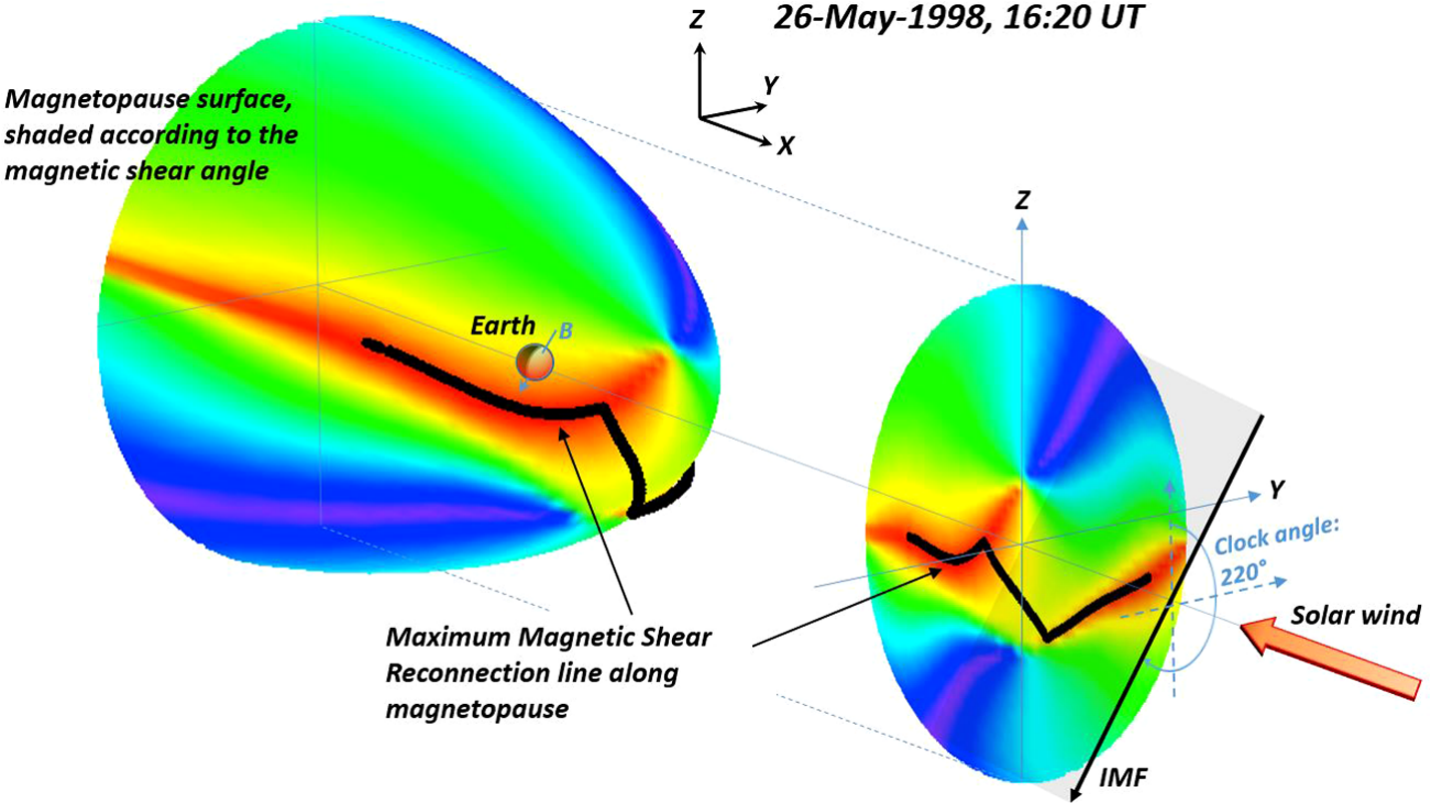
3D representation and 2D projection along the Sun-Earth line of the magnetopause surface, colored according to the magnetic shear angle. A maximum magnetic shear reconnection line across the dayside magnetopause is also shown (black). Adapted from Trattner et al. (2007)

An empirical model of the location of the reconnection line along the magnetopause surface for southward IMF conditions has recently been derived from detailed examinations of ion flux distributions in the high-altitude cusps (Trattner et al. 2007). The time-of flight characteristics of cusp ion distributions are used to determine velocity cutoffs for ions that precipitate from the magnetopause reconnection site and mirrored in the low altitude ionosphere (Onsager et al. 1990). In conjunction with magnetic field mapping techniques, this study resulted in the creation of a magnetopause reconnection location model that combines several of the features of the above-described X-line location concepts, and has come to be known as the Maximum Magnetic Shear model (e.g., Trattner et al. 2007). When the IMF is strongly southward ($155^{\circ} < \tan^{-1}(\text{B}_{\text{Y}}/\text{B}_{\text{Z}}) < 205^{\circ}$) or has a dominant B_X_-component ($|\text{B}_{\text{X}}|/\text{B}_{\text{TOT}} > 0.7$), this model predicts that steady dayside magnetic reconnection occurs along the two anti-parallel branches as described by Crooker (1979) and Luhmann et al. (1984). However, for other southward IMF conditions, magnetic reconnection is predicted to occur along a single contiguous X-line along the dayside magnetopause. This extended reconnection line does not necessarily pass through the subsolar standoff location. Rather, it occurs along a ridge of maximum magnetic shear between the opposing magnetic fields on the two sides of the magnetopause. This ridge of maximum magnetic shear is dependent on the dipole tilt angle, moving south of the equatorial plane during the northern summer, and north of the equatorial plane during northern winter. An example of the maximum magnetic shear reconnection line is shown in Fig. 4. Later chapters present observations and empirical study results that provide quantitative support for this model.

## Evidence for Reconnection at the Magnetopause and the Formation of Associated Boundary Layers

Although magnetic reconnection has been demonstrated to be the primary means of interconnection between the solar wind and magnetospheric plasmas, this understanding evolved over many years – as spacecraft instrumentation matured and in situ observations at higher spatial and temporal resolution were obtained. Indirect evidence of magnetopause reconnection (Aubry et al. 1970) was presented in the form of a decreasing geocentric distance to the magnetopause during extended periods of southward IMF and steady solar wind pressure. More direct evidence of reconnection was provided a decade later as the temporal resolution of the plasma measurements improved (e.g., Sonnerup et al. 1981).

Because of the magnetic reconnection process at the magnetopause, newly opened magnetic field lines rapidly move from the reconnection site with a speed related to the local Alfvén speed, tangent to the magnetopause surface. Accelerated ions from the reconnection site (also described as ion beams or ion jets) travel along the reconnected magnetic field lines, tangential to the magnetopause. These ions can be observed in situ by sampling spacecraft and provide information about the location of the reconnection site with respect to the spacecraft location.

The reconnection process during intervals of southward IMF mixes the plasma populations of the magnetosheath and the magnetosphere, and this mixed population is observed as thick (several hundred to a couple thousand km) boundary layers on either side of the thinner magnetic field rotation of the magnetopause current layer. The outer boundary layer (outside the magnetopause) is the magnetosheath boundary layer (MSBL), while the inner layer is called the low-latitude boundary layer (LLBL) because it was first identified at low latitude. The observed plasma within the boundary layers is a mixture of incident, reflected, and transmitted components (Cowley 1982). Most often, the magnetosheath population is significantly denser and has lower energy than that of the magnetosphere, and these differences in the plasma moments are readily observed within the boundary layers, enabling different components related to each source region to be easily identified via ion flux distribution functions.

Within the MSBL, heated field-aligned electrons are also observed streaming away from the reconnection line. While these in situ observations provide additional evidence of the occurrence of magnetopause reconnection and the general relative location of the spacecraft from the reconnection location, the heated electrons travel too fast to provide precise, quantitative estimates of the distance from the reconnection site at the Earth’s magnetopause. However, the directionality of electron signatures is often used to provide context with regard to whether a particular field line is open (connecting the magnetosphere to the solar wind) or closed (with both ends of the magnetic field tied to the ionosphere) (Fuselier et al. 2011, 2014a; Vines et al. 2015). Heated electrons have also been utilized in studies of the Cassini spacecraft in relation to reconnection sites at the significantly larger Saturnian magnetopause (Fuselier et al. 2014b, 2020) and are described in a later section.

**Fig. 5:**
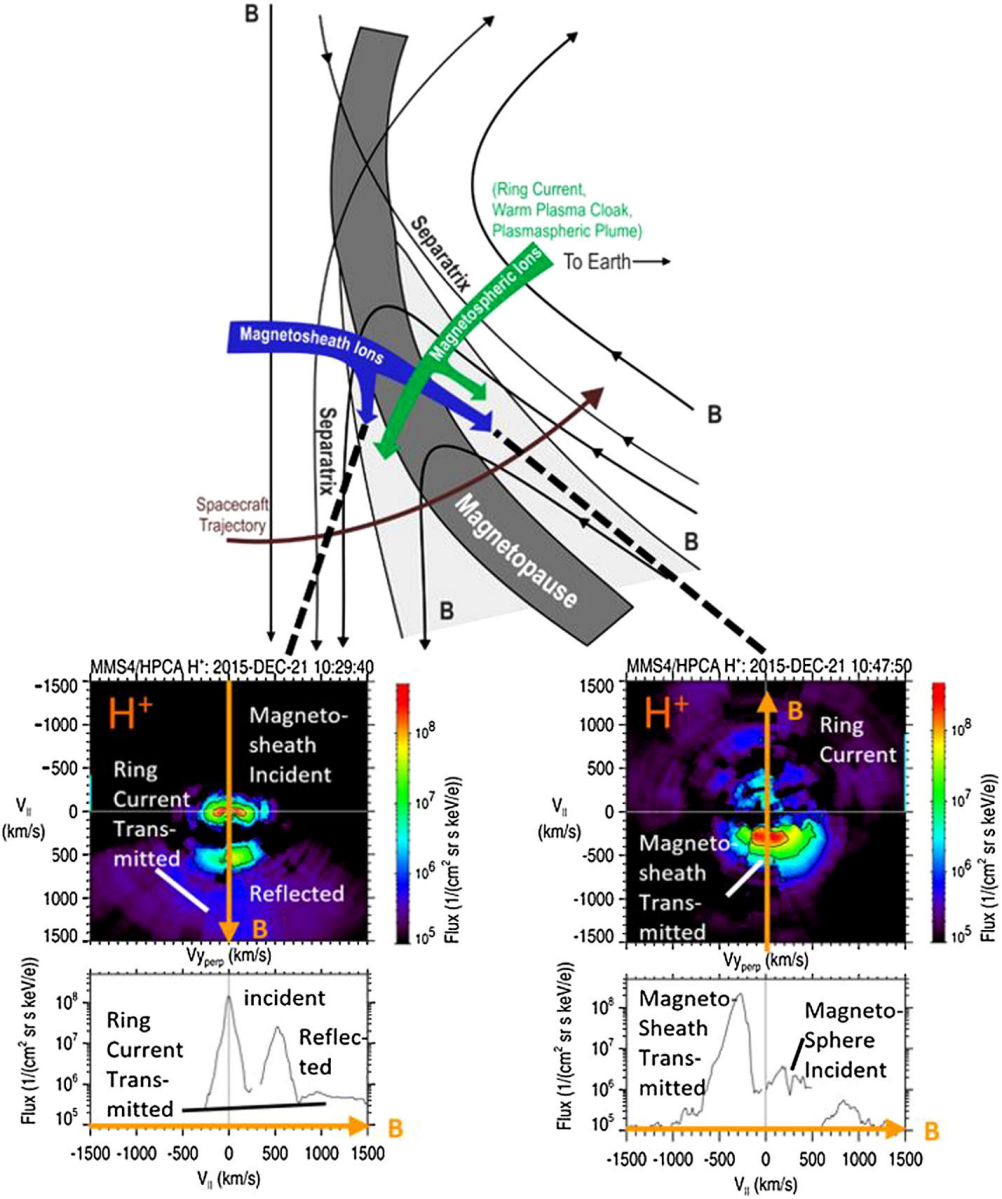
Top: A schematic magnetopause current layer and boundary layers as viewed in profile, including an active reconnection site somewhere northward of (above) the sampling spacecraft. Transmitted and reflected plasma component populations from the magnetosheath and magnetosphere are indicated. Bottom: 2D and 1D cuts of plasma distribution functions from MMS HPCA during encounters with these boundary layers

As an example, Fig. 5 illustrates distinct ion components during an inbound dayside, low-latitude magnetopause crossing by one of the spacecraft of the Magnetospheric Multiscale (MMS) constellation. At this time, the IMF was directed southward, and the MMS spacecraft crossed the magnetopause somewhere southward of the reconnection site (top panel). The magnetosheath (shocked solar wind) region is to the left, and the magnetosphere region is to the right. The separatrices denote the topological separation between the plasmas which have not yet undergone reconnection from the mixed plasma of the boundary layer which has recently undergone reconnection. The MSBL is represented by the light gray region left of and adjacent to the magnetopause, and the LLBL is represented by the light gray region to the right of the magnetopause. The bottom left panels of Fig. 5 illustrate cuts (2D and 1D) from an observed 3D proton flux distribution in the MSBL, observed by the Hot Plasma Composition Analyzer (HPCA). The distribution is shown in a magnetic field-aligned coordinate system (represented by the downward directed B-vector) over a 10-second interval (1/2-spin period of MMS). The proton populations observed include a nearly stagnant incident magnetosheath component, a magnetosheath component that is reflected at the magnetopause X-line, and a low-flux, high-energy component representing transmitted protons from the magnetosphere ring current. The bottom left panel shows the 1D cut of flux measurements in the MSBL along the magnetic field. The proton flows are directed parallel to the magnetic field, consistent with a reconnection region at some location northward of (above) the spacecraft. Such observations of accelerated ions provide strong evidence that magnetic reconnection has recently occurred (or continues to occur) at the magnetopause somewhere along the sampled magnetic field line (Trenchi et al. 2008; Trattner et al. 2012a; Petrinec et al. 2016).

The bottom right panels of Fig. 5 illustrate 2D and 1D cuts of the proton flux distribution within the LLBL observed by MMS/HPCA. The largest flux is from the transmitted magnetosheath plasma through the reconnection site and is observed moving anti-parallel to the magnetospheric magnetic field, again consistent with a reconnection region northward of (above) the spacecraft. Incident magnetosphere and ring current protons are also observed, at much reduced flux as compared to the magnetosheath component. Again, while such observations indicate the general location of magnetic reconnection in relation to the sampling spacecraft (i.e., northward or southward), the exact reconnection site location is not revealed.

**Fig. 6:**
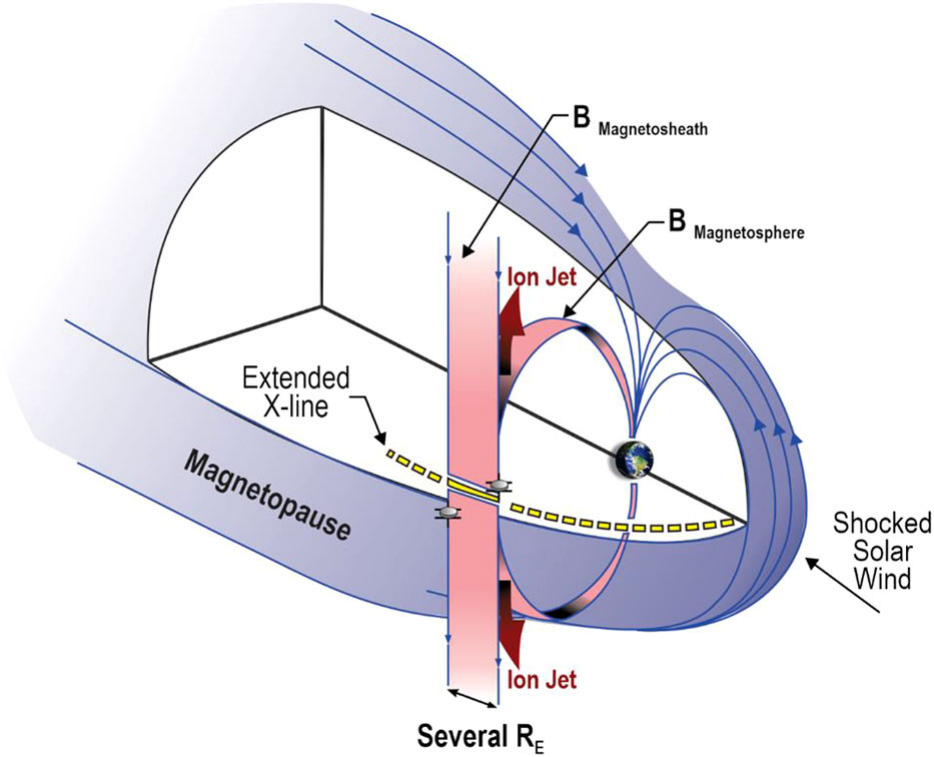
A schematic of the 3D magnetopause, including a low-latitude reconnection line during an interval of strongly southward IMF. Ion jets emanating from the reconnection line and tangential to the magnetopause are shown. Adapted from Phan et al. (2000)

Occasionally, simultaneous observations from multiple spacecraft can be used to constrain the location of magnetic reconnection. Figure 6 shows a scenario where two spacecraft at the magnetopause simultaneously straddle the reconnection X-line. The spacecraft above the X-line would observe a jet propagating parallel to the magnetic field in the LLBL, while the spacecraft below the X-line would observe a jet propagating anti-parallel to the magnetic field in the LLBL. The simultaneous observation of reconnection at the magnetopause by two well-separated spacecraft is expected to be a rare event. However, the scenario depicted in Fig. 6 is adapted from actual observations in Phan et al. (2000). For this fortuitous occurrence, oppositely directed jets along the flank magnetopause were observed with two spacecraft: Equator-S and Geotail.

**Fig. 7:**
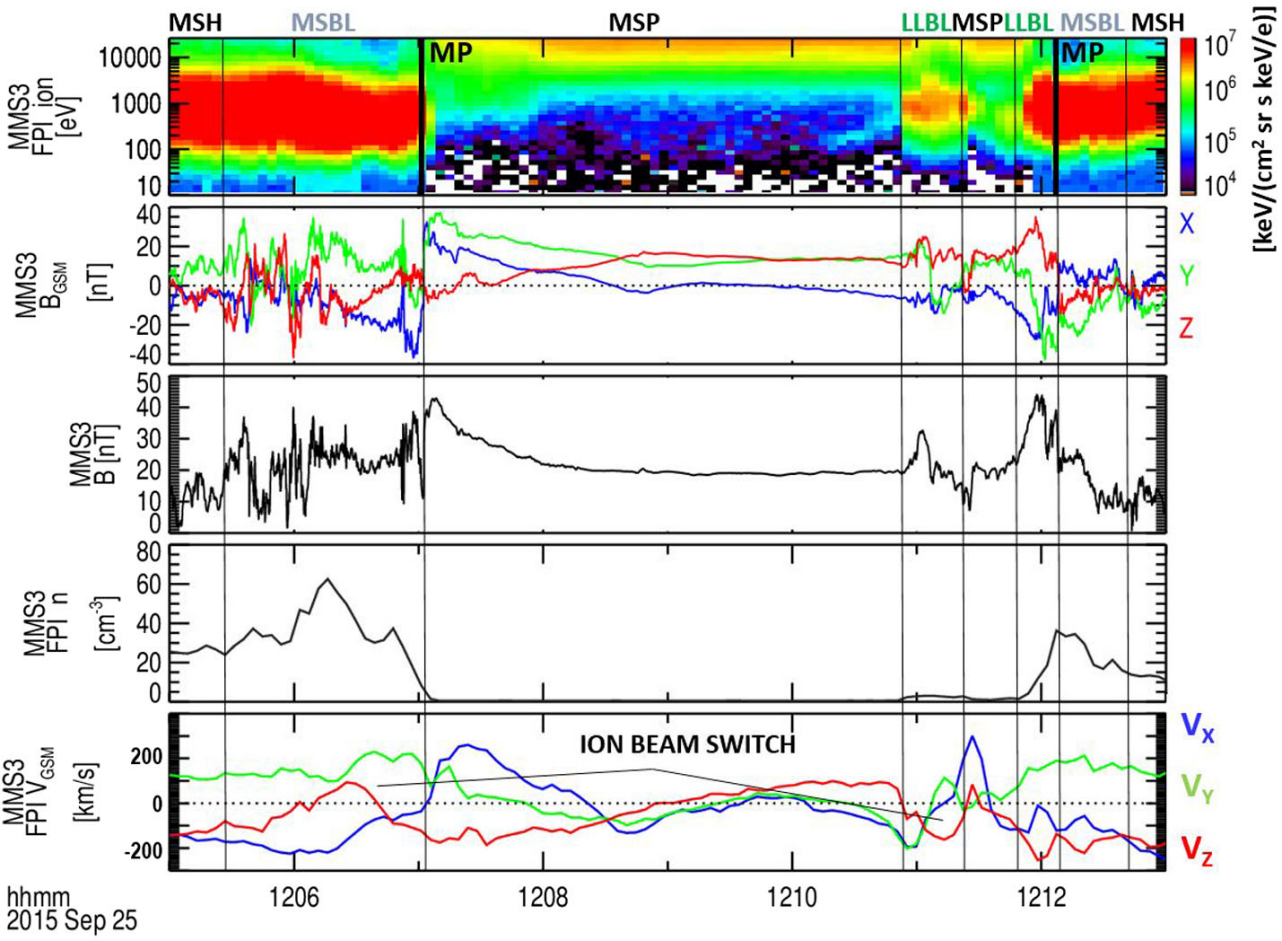
Two distinct magnetopause crossings and surrounding boundary layers observed on 25 September 2015 by the MMS spacecraft. A switching ion beam (depicted by a large reversal in the ion $\text{V}_{\mathrm{z}}$ component) was observed at $\sim12{:}07~\text{UT}$ in the magnetopause boundary layer. Adapted from Trattner et al. (2017a)

Ion beam switches tangent to the magnetopause observed by a single spacecraft should be more numerous than simultaneous observations from two well-separated spacecraft at the magnetopause. These ion beam switches indicate that the reconnection site passed by the spacecraft. An ion beam switch, observed by MMS3 in the high latitude southern dusk sector, is illustrated by two distinct magnetopause crossings in Fig. 7. The first of the two magnetopause crossings includes an example of an ion jet reversal (a large reversal of the $\text{V}_{\text{Z}}$ component of the ion velocity within the boundary layers) as presented in the bottom panel (from Trattner et al. 2017a). The spacecraft did not necessarily enter the very small reconnection diffusion region (where the electrons and ions are unmagnetized and the magnetic fields reconnect), but during the beam switch, the spacecraft had to be very close to the reconnection site.

While these observations demonstrate the presence and location of magnetic reconnection at the magnetopause, they provide very limited information on the large-scale extent of the process, as well as limited information regarding the detailed features of this process. The basic problem is that spacecraft do not reside at the magnetopause for very long, and the reconnection X-line occupies a very small amount of the total surface area of the magnetopause. Therefore, despite a large number of magnetopause crossings from many spacecraft missions, the number of crossings where the reconnection site is pinpointed on the magnetopause is too small to develop a statistical description of the location of the reconnection X-line for the wide range of solar wind conditions. Some of these characteristics are described in the following chapters.

## The Length of the Magnetopause X-Line

### Evidence for Long X-Lines at the Magnetopause: In Situ Observations at the Magnetopause

Most early models of reconnection at the dayside magnetopause assumed that there is a long, quasi-continuous X-line at the boundary. Notable exceptions are the Flux Transfer Event (FTE) concept of reconnection localized in both time and space (Russell and Elphic 1978, 1979) and the postulation that the low-latitude boundary layer may be produced by random reconnection at the magnetopause for northward IMF (Nishida 1989). In situ evidence for long, quasi-continuous X-lines at the magnetopause are limited because single spacecraft cross the boundary at a single location and at a single time. Simultaneous direct evidence of extended reconnection lines requires simultaneous in situ observations from multiple locations (e.g., Peterson et al. 1998; Phan et al. 2000). For example, simultaneous observations of reconnection from two widely spaced spacecraft at the flank magnetopause was consistent with an X-line that was at least $3~\text{R}_{\text{E}}$ long (see Fig. 6). In another example during a southward IMF interval, Dunlop et al. (2011) used a 10-spacecraft conjunction at the magnetopause to show that the reconnection X-line may have extended over nearly the entire dayside magnetopause.

### Evidence for Long X-Lines at the Magnetopause: Remote Sensing Observations

Remote sensing observations of magnetic reconnection, particularly from the magnetospheric cusps, are the primary observations that provide evidence for long, quasi-continuous X-lines at the magnetopause. Magnetosheath ions entering the dayside magnetopause precipitate into the magnetospheric cusps. By identifying the location of these precipitating ions in thousands of cusp crossings by e.g., the Polar and Cluster spacecraft (covering all dayside local time sectors), and tracing the field lines back to the magnetopause using a magnetospheric field line model, the extent of the reconnection X-line is determined. In situ observations of ion precipitation from spacecraft crossing the cusp are used to determine the distance to the reconnection X-line. The details of this procedure are described in the next chapter. Spacecraft almost never follow a single reconnected flux tube as they traverse the cusp. Instead, they cross different flux tubes and, by tracing these flux tubes back to the magnetopause, the extent of the X-line is determined.

Another technique for determining the X-line length is to use global auroral imagers, particularly proton aurora imagers (e.g., Fuselier et al. 2002). Imaging the precipitation of energetic ($>2~\text{keV}$) protons produces a map in the ionosphere of the reconnecting flux tubes that not only provides the local time extent of the X-line, but also the time variability of reconnection. In one example for northward IMF, the precipitation was observed continuously (with 2-min time resolution) for hours, indicating that reconnection was quasi-steady for a very long time (Frey et al. 2003a,b). In another northward IMF example, the reconnection X-line was estimated to have a length of about $5~\text{R}_{\text{E}}$, extending largely tailward, poleward of the cusp (Fuselier et al. 2002). For two other southward IMF examples, the reconnection X-line was estimated to be $20\text{--}25~\text{R}_{\text{E}}$ and $10~\text{R}_{\text{E}}$ long, respectively. That is, it was estimated to extend essentially over the entire dayside magnetopause.

Images of the proton aurora on the dayside also help distinguish between component and anti-parallel reconnection. As discussed in the introduction, component reconnection produces an X-line that is tilted at an angle with respect to the GSM equator at the local noon meridian. In contrast, anti-parallel reconnection produces two X-lines that extend from the cusp to the dawn and dusk terminators (see Fig. 2). These two types of X-lines produce different ionospheric precipitation patterns for $>2~\text{keV}$ protons accelerated in the reconnection process.

**Fig. 8:**
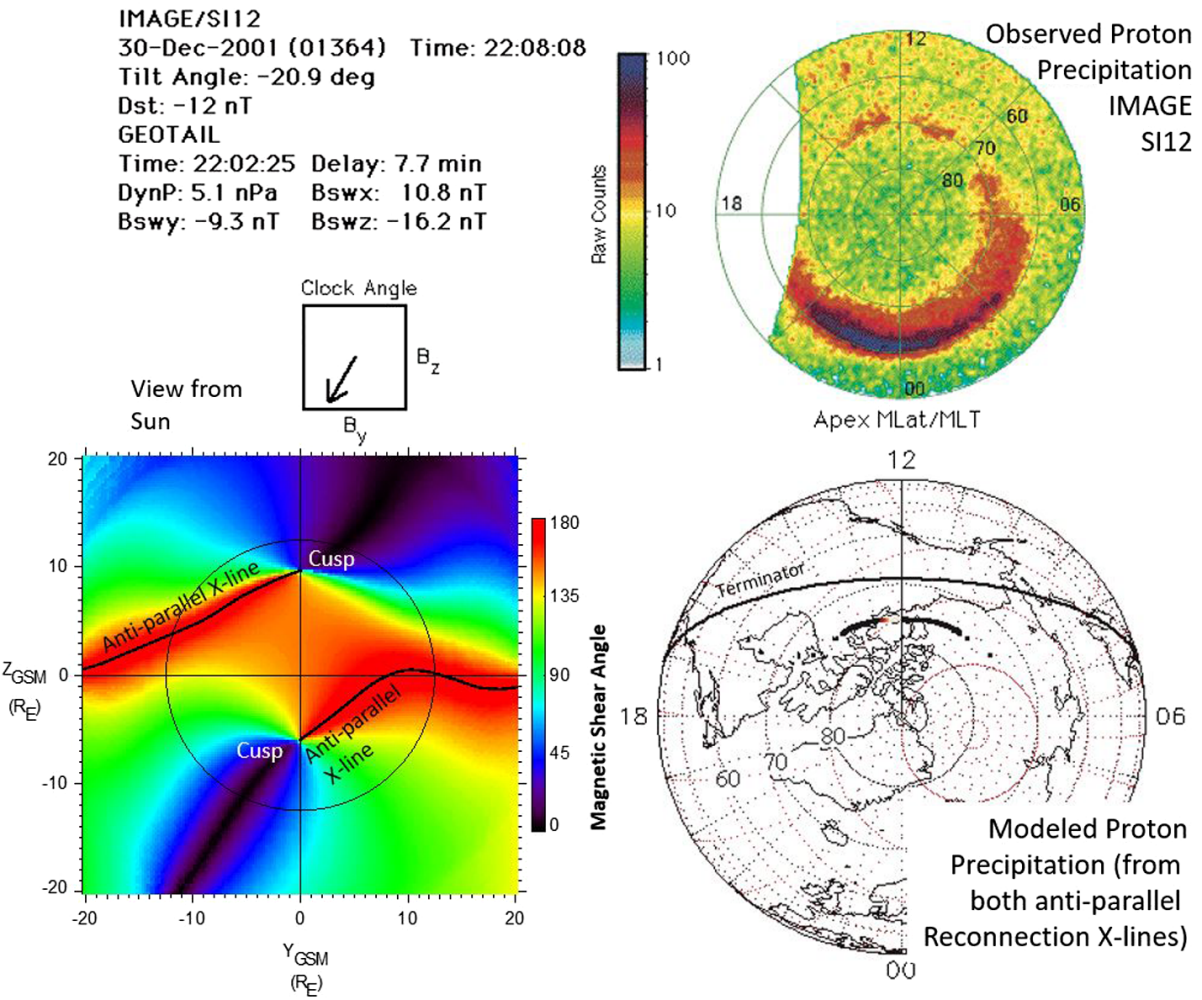
Predicted and observed cusp proton precipitation for a strongly southward IMF interval. The lower left-hand plot shows the modeled magnetic shear at the magnetopause for these IMF conditions. The view is from the Sun and the shear is projected on the Y–Z GSM plane. The thin black line is the terminator. Two anti-parallel X-lines extending from the northern and southern cusps are predicted. The right-hand panels show the observed (top panel) and predicted proton precipitation (bottom panel) for the reconnection X-lines shown in the lower left. The predicted and observed precipitation patterns are very similar, indicating that there are two X-lines that extend across the entire dayside magnetopause

Figure 8 shows an example of how the different precipitation patterns provide information on the extent of the X-line and helps distinguish component and anti-parallel reconnection (Petrinec and Fuselier 2003). In the top left-hand part of the figure, the date, time, Dst, and solar wind conditions (from the Geotail spacecraft) are shown. The IMF was strongly southward with a clock angle of $210^{\circ}$. The bottom left-hand part of the figure shows the magnetic shear angles at the magnetopause projected onto the Y–Z GSM plane. In this panel, red shows high shear and black is low shear. For the IMF clock angle, two anti-parallel reconnection X-lines are predicted to extend toward the magnetospheric flanks from the northern and southern hemisphere cusps at noon local time ($\text{Y}_{\text{GSM}} = 0$).

The bottom right-hand panel of Fig. 8 shows the modeled precipitation pattern from the two anti-parallel reconnection X-lines in the bottom left. A relatively thin region of precipitation centered on local noon but with a gap in the duskside ionosphere is predicted. The gap is produced because protons accelerated in the reconnection process near the reconnection X-line immediately duskward of noon local time, originate in the southern hemisphere and must propagate against the southward magnetosheath flow to precipitate in the northern cusp. Protons propagating against the magnetosheath flow lose energy and precipitate with energies $<2~\text{keV}$, that is, below the energy threshold of the auroral imager. The top right-hand panel in Fig. 8 is the proton aurora image from the IMAGE/SI12 imager (Mende et al. 2000). The predicted and observed precipitation pattern compare well, indicating that there are two X-lines extending from the two cusps and that the reconnection X-lines extend over the entire dayside magnetopause.

## The Magnetospheric Cusps and the Location of the X-Line

As described in the previous sections, the most direct way to determine the location of the magnetic reconnection line are in situ observations at the Earth’s magnetopause. Observers identify events where accelerated ion beams in the magnetopause boundary layers, emanating from the X-line, switch direction (e.g., Cowley 1982; Gosling et al. 1982; Trenchi et al. 2008; Dunlop et al. 2011), or identify events with the typical signatures of an ion diffusion region (IDR) or an electron diffusion region (EDR) (e.g., Burch et al. 2016). Since the target area is very small compared to the size of the magnetopause, this methodology is challenging but not impossible (e.g., Fuselier et al. 2017; Webster et al. 2018). However, the number of magnetopause crossings that yield the location of the reconnection line is only of the order of a few percent of the total number of crossings for a particular spacecraft mission. Thus, even very large data sets containing thousands of magnetopause crossings yield only a few tens of crossings where the location is determined.

Alternatively, an excellent magnetospheric region to study magnetic reconnection, including the location of the reconnection line, reconnection variability and reconnection rate, are the magnetospheric cusps. The magnetospheric cusps are narrow funnel-shape regions through which the shocked solar wind plasma has direct access to the magnetosphere and the ionosphere, making the cusps one of the denser plasma regions in the magnetosphere. Despite their small size and small ionospheric footprints, the cusps play an important role in the transfer of plasma, energy, and momentum from the solar wind to the magnetosphere. Since all magnetic field lines that reach the magnetopause converge into the cusp area, every process that occurs on the magnetopause, including reconnection at the magnetopause, leaves a signature in the precipitating ion profile, making the cusp a very compact and versatile region for study. As it will become clear later in this chapter, the reconnection signature in the cusp allows determination of the distance to the reconnection line multiple times during a cusp crossing. Thus, unlike a magnetopause crossing, nearly all cusp crossings provide many independent measurements of the location of the reconnection line.

The cusps, first observed by Heikkila and Winningham (1971) and Frank (1971), are also one of the most dynamic regions in the magnetosphere. The cusp locations are sensitive to external conditions, e.g. the IMF B_z_ component (Burch 1973), the solar wind dynamic pressure, and IMF intensity (e.g., Carbary and Meng 1986; Newell et al. 1989; Escoubet and Bosqued 1989; Woch and Lundin 1992; Pitout et al. 2006). The magnetic latitudes of the cusps are also dependent on the Earth’s dipole tilt angle (e.g., Zhang et al. 2005).

Once reconnection occurs somewhere at the magnetopause, magnetosheath ions stream continuously along the newly opened magnetic field lines from the magnetosheath into the cusp and subsequently into the magnetotail (e.g., Lockwood and Smith 1993, 1994; Onsager et al. 1993). Simultaneously, the newly opened magnetic field lines convect with the solar wind, causing very distinctive ion energy dispersions in the cusp, a velocity filter effect with lower-energy ions convecting further poleward than higher-energy ions. Such a velocity dispersion was predicted for southward directed IMF conditions (Rosenbauer et al. 1975; Reiff et al. 1977) and observed by Shelley et al. (1976).

**Fig. 9:**
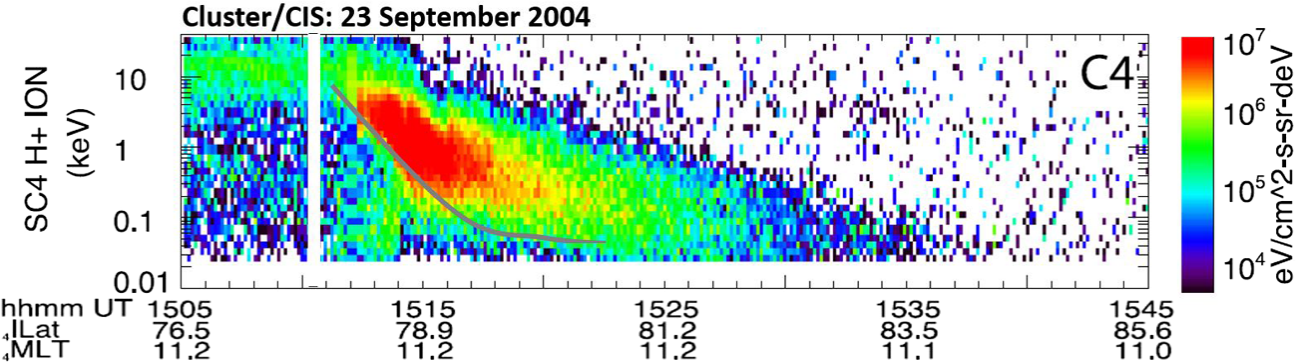
Omnidirectional $\text{H}^{+}$ energy flux observed by Cluster/CIS4 on 23 September 2004 in Earth’s northern cusp region (adapted from Fig. 3 in Escoubet et al. 2008). The precipitating magnetosheath ions exhibit the typical cusp ion velocity dispersion, with lower-velocity ions arriving at higher latitudes due to the convection of newly opened magnetic field lines

Figure 9 shows omnidirectional proton energy flux observed by Cluster/CIS4 on 23 September 2004 in Earth’s northern cusp region (Escoubet et al. 2008). The spacecraft encounters precipitating magnetosheath ions with energy of about 10 keV as it crosses the open-closed field line boundary at 15:11 UT. As Cluster/CIS4 progresses to higher latitudes away from the open-closed field line boundary, the energy of the precipitating ion smoothly decreases as lower-energy ions from the dayside reconnection site arrive at higher latitudes, exhibiting the typical cusp ion-velocity dispersion.

For the majority of cusp crossings, the decrease of the precipitating ion velocity with increasing latitude is generally not smooth, but shows complicated structures with variations in flux levels and sudden changes in the energy of the precipitating ions (e.g., Newell and Meng 1991; Escoubet et al. 1992). Such variations in the cusp ion energy dispersion profile are known as “stepped” or “staircase” cusp ion signatures. These cusp structures have been predicted by Cowley et al. (1991) and Smith et al. (1992), based on a model by Cowley and Lockwood (1992) describing the temporal nature of the magnetopause reconnection process. In this pulsating cusp model (Lockwood and Smith 1989, 1990), steps are the result of changes in the reconnection rate at the magnetopause that create neighboring flux tubes in the cusp with different time histories since reconnection (e.g., Lockwood and Smith 1994).

An important feature of temporal cusp steps is their convection with the open magnetic field lines under the joint action of magnetic tension and shocked solar wind flow, creating an ever-changing structural profile of precipitating ions in the cusp. In addition to the temporal nature of the magnetic reconnection process, cusp steps have also been reported to be caused by spatially separated reconnection lines at different local times across the magnetopause (e.g., Weiss et al. 1995; Onsager et al. 1995; Trattner et al. 2005, 2008).

To determine the location of the dayside reconnection line from cusp ion observations, the time-of-flight characteristics of the precipitating and mirrored magnetosheath ions in the cusp are used. A spacecraft located in the high-altitude cusp region observes magnetosheath ions arriving direct from the magnetopause reconnection site (incident ion beam) and magnetosheath ions that first traveled downward to the ionosphere, mirrored there, and then traveled upward, returning to the altitude of the cusp-observing spacecraft (mirrored ion beam). Since both the incident and mirrored ion beam are on the same reconnected field line, they experience the same time history of reconnection, therefore, the determination of the location of the dayside reconnection site described below is not affected by temporal or spatial changes in the reconnection rate.

**Fig. 10:**
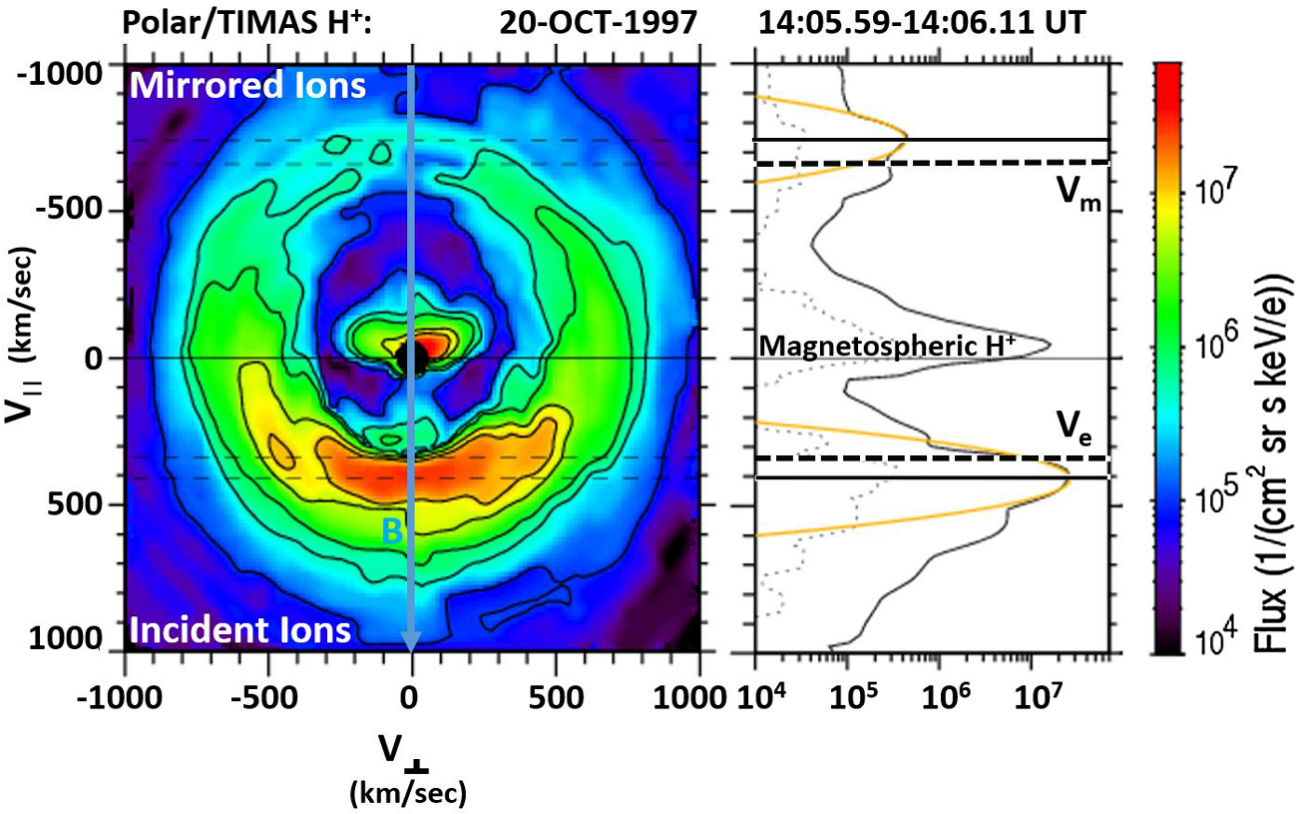
2D cut of the 3D distribution observed by Polar/TIMAS, showing (left panel) the velocity space distribution in a plane containing the magnetic field (X-axis), and (right panel) the 1D cut of the distribution along the magnetic field direction showing the incident and mirrored ion beams with the cutoff velocities Vm and Ve

Figure 10 shows a cusp proton distribution observed by the Toroidal Imaging Mass Angle Spectrometer (TIMAS) instrument on board the Polar spacecraft during a cusp crossing on 20 October 1997, from 14:05.59 UT to 14:06.11 UT. The proton velocity distribution is plotted in magnetic field-aligned coordinates, with the bulk velocity perpendicular to the magnetic field removed. The ambient magnetic field direction in the left panel of Fig. 10 is along the vertical axis pointing downward, showing the distribution for the precipitating incident magnetosheath ions injected at the magnetopause reconnection site as well as the mirrored ions returning from the ionospheric mirror points.

The right panel of Fig. 10 shows a cut through the cusp proton distribution in the left panel along the ambient magnetic field direction. The peaks for the incident and mirrored ion beams are fit with Gaussian distributions (orange curves) for consistency in determining the cutoff velocities. These ion beam cutoff velocities are used to determine the distance between the cusp spacecraft and the reconnection site at the magnetopause. The methodology is generally known as the low-velocity cutoff method and was first used by Onsager et al. (1990, 1991) in the Earth’s plasma sheet boundary layer to estimate the distance to the reconnection site in the magnetotail. However, in that region of the magnetosphere, the observing spacecraft is usually close to the nightside ionospheric mirror site and far away from the magnetotail reconnection site, resulting in large uncertainties in the distance estimate. On the other hand, calculating the distance to the reconnection site using this methodology works very well for high-altitude cusp spacecraft observations since this location is roughly half-way between the mirror point in the ionosphere and the magnetopause reconnection location (e.g., Fuselier et al. 2000; Trattner et al. 2007, 2012b).

The cutoff velocities for the incident (Ve) and mirrored (Vm) ion beams in the cusp distributions in Fig. 10 are defined at the low-speed side of each ion distribution where the flux is a factor of 1/e lower than the peak flux (see also Fuselier et al. 2000; Trattner et al. 2005, 2007). The cutoffs are marked with black dashed lines in Fig. 10.

**Fig. 11:**
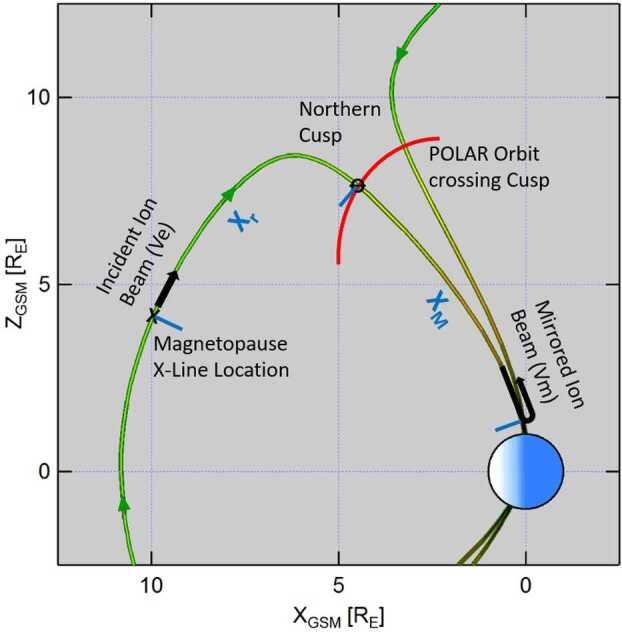
The Polar satellite, crossing the northern cusp region, observes precipitating ions injected onto newly opened magnetic field lines at the dayside magnetopause X-line and ions returning from the low-altitude mirror points. The specific cutoff velocities Ve and Vm in the incident and mirrored ion beams, respectively, are used to determine the distance from the Polar satellite to the dayside X-line Xr

Figure 11 shows a schematic drawing of the low-velocity cutoff method with the incident and mirrored ion beams in the Earth magnetospheric cusps. The equation to determine the distance to the magnetopause reconnection site, X_r_, is:

1 $$ \text{Xr/Xm} = 2 \text{Ve}/(\text{Vm} - \text{Ve}) $$

where Xm is the distance to the ionospheric mirror point (e.g., Onsager et al. 1990; Fuselier et al. 2000). The distance to the mirror point, Xm, is determined by using the position of the spacecraft in the cusp and tracing the geomagnetic field line from this position down to the ionosphere using the T96 model (Tsyganenko 1995).

Plasma observations in the cusp, together with the calculated distance to the reconnection site Xr from Eq. (1) and the known cutoff speed of the precipitating and mirrored ion distributions Ve and Vm, are also used to determine how long the present cusp magnetic field line has been open. This is the “time since reconnection” ($\Delta t$): the elapsed time from the moment that the ions are injected at the reconnection site and the current time of observed plasma on a reconnected field line.

2 $$ \Delta t = \text{Xr} / \text{Ve} = 2\text{Xm} / (\text{Vm} - \text{Ve}) $$

The term “time since reconnection” was also used in modeling cusp dispersions and studies of FTEs (see, e.g., Lockwood and Hapgood 1998). However, with this combined methodology, the temporal evolution of open cusp magnetic field lines throughout the cusp is known. In addition, the location of the reconnection site at the magnetopause is known, and, through magnetic field models, local conditions (magnetic shear) at the magnetopause reconnection site.

The distance to the reconnection line Xr is subsequently traced back to the magnetopause, starting at the location of the spacecraft in the cusp and again using the T96 model. The end points of these field line traces mark the entry points of magnetosheath plasma into the magnetosphere, the dayside reconnection location.

## The Maximum Magnetic Shear Model

The low-velocity cutoff method described in the previous chapter was used in a study of 130 crossings of the northern cusp region by the Polar spacecraft (Trattner et al. 2007). The study used observations from the Polar/TIMAS instrument and was designed to determine the dayside magnetic reconnection location for a broad range of solar wind and southward IMF conditions. The study is the basis for the development of the Maximum Magnetic Shear model.

**Fig. 12:**
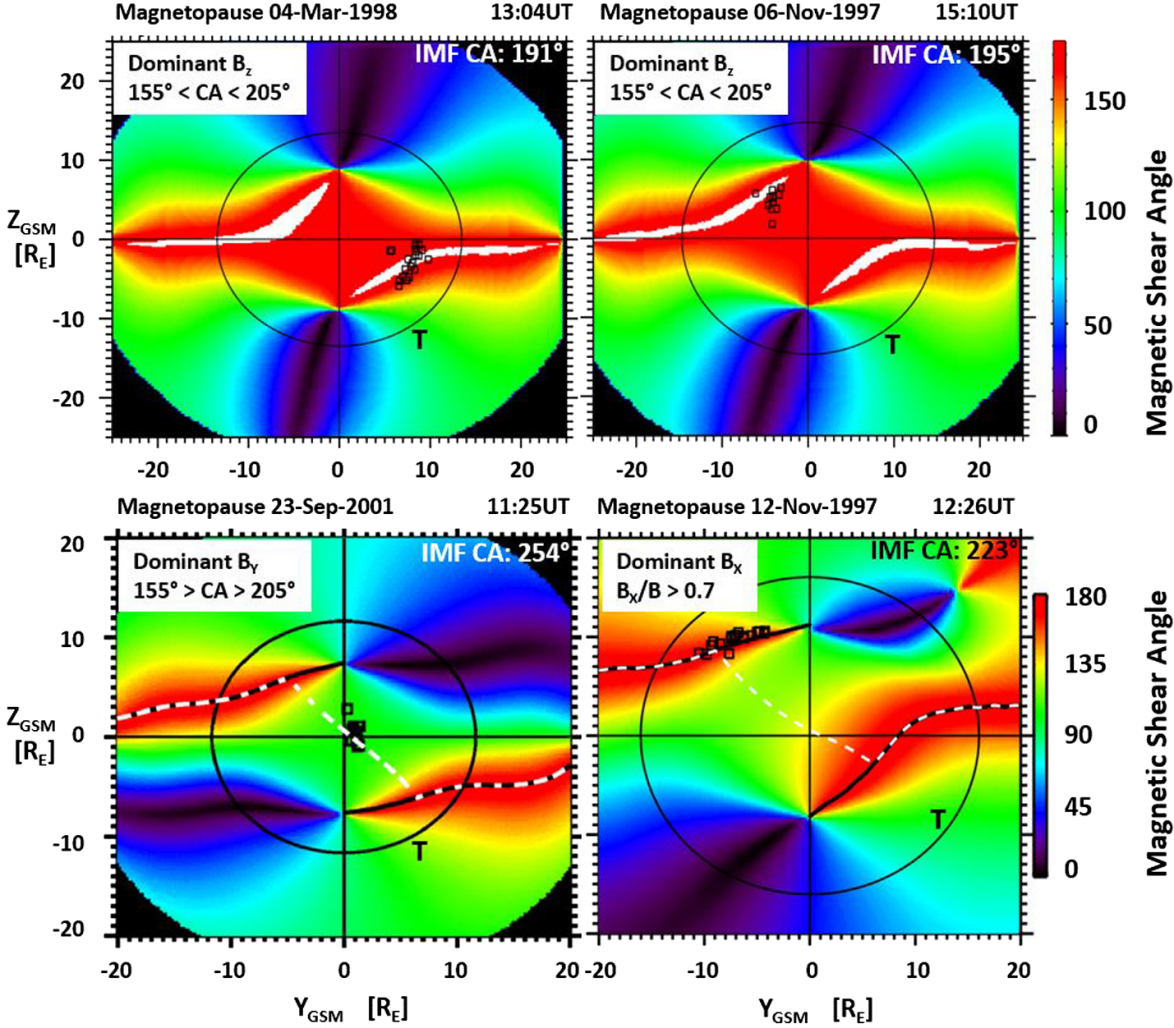
The magnetic shear angle across the dayside magnetopause, as seen from the Sun, for various IMF conditions. The circle represents the magnetopause shape at the terminator plane. Black squares represent the locations of the X-line at the magnetopause, determined by the low-velocity cutoff method. The methodology led to the development of an empirical prediction model for the dayside location of the X-line, the Maximum Magnetic Shear model. Top panels show anti-parallel reconnection for dominant IMF B_Z_ conditions. Bottom panels show the component reconnection scenario for dominant IMF B_Y_ conditions with an X-line along the locus of maximum magnetic shear across the magnetopause (left) and anti-parallel reconnection for dominant IMF B_X_ conditions (right)

To visualize the dayside magnetopause reconnection location, using the end points from the magnetic field traces that originated at the spacecraft location in the cusp, a magnetic shear angle plot for the dayside magnetopause is used (see also Figs. 4 and 8). Figure 12 shows four examples of magnetopause magnetic shear angle plots projected onto the Y–Z_GSM_ plane from the original cusp study (Trattner et al. 2007).

The magnetopause magnetic shear angles are determined using the convected solar wind input conditions and internal and external magnetic field models. For the internal magnetic field model, the T96 model (Tsyganenko 1995) is used, while the external model is represented by the analytic Kobel and Flückiger (1994) magnetic field draping model. To determine the magnetopause magnetic shear for the state before magnetic reconnection occurs, only closed model field lines from the T96 model on the magnetospheric side of the Sibeck et al. (1991) magnetopause are selected. Details on determining the magnetic shear at the magnetopause are described in Trattner et al. (2007).

Red areas in the magnetic shear angle plots represent the magnetopause anti-parallel reconnection regions where the internal and external magnetic fields are nearly anti-parallel (i.e., magnetic shear angles $>160^{\circ}$), with white areas representing regions where the model magnetic fields are within $3^{\circ}$ of being exactly anti-parallel. The black circles in Fig. 12 represent the shape of the magnetopause at the terminator plane, while the black symbols are the end points from the cusp magnetic field line traces, representing the plasma entry points at the magnetopause for the precipitating ions observed in the cusp. The white dashed line crossing the dayside magnetopause in the bottom panels of Fig. 12 represents the location of the predicted component reconnection tilted X-line based on predictions from the Maximum Magnetic Shear model. The Maximum Magnetic Shear model predicts long X-lines extending across the entire dayside magnetopause (e.g., Fuselier et al. 2002; Phan et al. 2006; Dunlop et al. 2011). In this model, the component reconnection tilted X-line segment connects the two anti-parallel reconnection regions on the dayside, and these anti-parallel reconnection regions continue along the flanks towards the magnetotail.

The top panels of Fig. 12 show the typical reconnection locations for dominant southward IMF B_Z_ conditions. For the cusp event observed on 4 March 1998 at 13:04 UT with an IMF clock angle of $191^{\circ}$, the plasma entry points are concentrated at the anti-parallel reconnection regions in the southern dusk sector (top left panel). For the cusp event observed on 6 November 1997 at 15:10 UT with an IMF clock angle of $195^{\circ}$, the plasma entry points also group around the anti-parallel reconnection region located in the northern dawn sector (top right panel). Using the Polar cusp survey of 130 events, it was determined that all cusp events for IMF conditions $\pm25^{\circ}$ of purely southward IMF exclusively map to the anti-parallel regions. That is, under dominant -B_Z_ conditions, there is anti-parallel reconnection across the dayside magnetopause in agreement with the southward IMF case observed by the IMAGE spacecraft and discussed in Fig. 8.

For cusp events observed during dominant IMF B_Y_ conditions, the plasma entry points group around a component reconnection tilted X-line located along the ridge of maximum magnetic shear across the dayside magnetopause. An example for these solar wind conditions is found in Fig. 12, bottom left panel. The cusp event observed on 23 September 2001 at 11:25 UT with an IMF clock angle of $254^{\circ}$ shows plasma entry points grouping at the subsolar point, which agrees with the location of the line of maximum magnetic shear. Therefore, for dominant IMF B_Y_ conditions, there is a mix of component and anti-parallel reconnection at the dayside magnetopause.

For cusp events observed during dominant IMF B_X_ conditions ($|\text{B}_{\text{X}}|/\text{B} > 0.7$), the plasma entry points again map to the anti-parallel reconnection region. An example for these input conditions is shown in the bottom right panel of Fig. 12. The cusp study concluded that nearly radial IMF conditions cause the draped IMF lines to first make contact with the magnetopause at high latitudes, where the maximum magnetic shear location is the anti-parallel reconnection site approaching the cusp regions. However, the limited number of events in this parameter range requires revisiting in future studies.

**Fig. 13:**
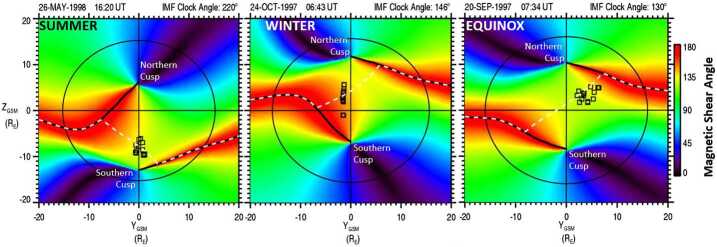
For dominant IMF B_Y_ conditions, the empirical Maximum Magnetic Shear model predicts a shift of the component reconnection tilted X-line with the seasons. Based on the magnetic field lines trace points from cusp observations (black symbols), the predicted reconnection location is shifted to the south during the northern hemisphere summer month (left panel), to the north during the winter month (middle panel) and only crosses the subsolar region during the equinoxes (right panel)

In the original cusp study (Trattner et al. 2007), cusp events observed during a dominant IMF B_Y_ component also revealed a strong dependence of the dayside reconnection location on the seasonal tilt of the dipole axis (see also Hoshi et al. 2018). This seasonal dependency for the dayside reconnection location at the Earth’s magnetopause is shown in Fig. 13, using examples for the northern hemisphere summer months (left panel), winter months (middle panel) and equinoxes (right panel). The format for the panels is the same as those in Fig. 12.

During the equinoxes (Fig. 13, right panel), the magnetic shear across the magnetosphere is symmetric with mirror images for the location of the anti-parallel reconnection region in the two hemispheres and both cusp locations at about the same distance from the equator. For such equinox cusp events, the field line trace points show that plasma entry locations at the magnetopause line up along a component reconnection tilted X-line that crosses the subsolar region. This reconnection location is closest to the location in the original proposed tilted X-line models, where X-lines are anchored in the subsolar region (e.g., Gonzalez and Mozer 1974; Sonnerup 1974). The observed component reconnection tilted X-line in Fig. 13 (right panel), observed on 20 September 1997 with an IMF clock angle of $130^{\circ}$, is also located at the ridge of maximum magnetic shear (dashed white line) across the dayside magnetopause and is in agreement with the predictions from the model.

For cusp events observed during the northern hemisphere summer months (Fig. 13, left panel), the low-velocity cutoff methodology consistently provided values for the distance to the reconnection site Xr that traces to locations south of a tilted X-line crossing the subsolar region. The left panel in Fig. 13 shows the magnetic shear across the magnetopause during the Polar cusp event observed on 26 May 1998 at 16:20 UT with an IMF clock angle of $220^{\circ}$. The field line trace points are located at local noon at high latitudes in the southern hemisphere, close to the location of the southern cusp region. As in previous events, the location is along the ridge of maximum magnetic shear across the dayside magnetopause as indicated by the dashed white line predicted by the model. This ridge of maximum magnetic shear crosses the noon meridian considerably south of the subsolar point.

For all cusp events observed during the northern hemisphere winter months (Fig. 13, middle panel), the field line trace points are consistently located north of the tilted X-line crossing the subsolar region. The middle panel of Fig. 13 shows that the trace points for the Polar cusp crossing on 24 October 1997 at 06:43 UT with an IMF clock angle of $145^{\circ}$ are located close to local noon in the northern hemisphere, centered around the line of maximum magnetic shear as predicted by the model.

A polar-orbiting spacecraft does not usually follow the same open, convecting flux tube as it traverses the cusp. Instead, it crosses neighboring open flux tubes as a result of two effects. First, the orbit trajectory passes through different local time sectors. Second, solar wind conditions with a significant IMF B_Y_ component modify the convection pattern to include a significant dawn-dusk component to the convection velocity. Applying the low-velocity cutoff method to orbits with an east-west component provides the location of the dayside magnetic reconnection location over a wide range of local times across the magnetopause over a short amount of time.

**Fig. 14:**
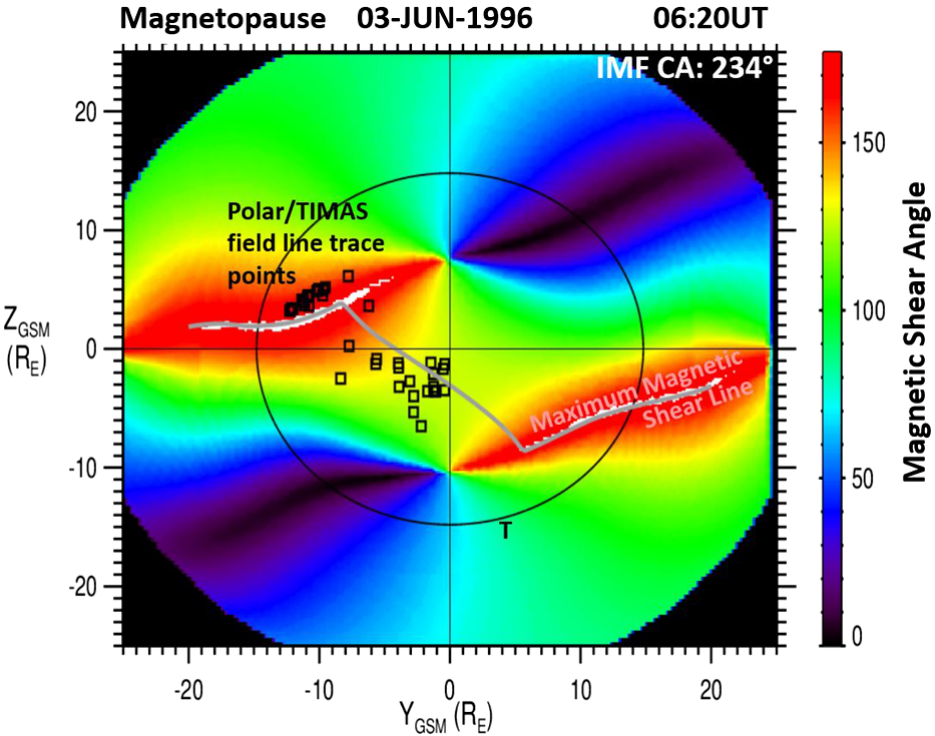
The magnetic shear across the magnetopause on 03-June-1996 at 06:20 UT. Black symbols represent the Polar/TIMAS field line trace points, determined by using the low-velocity cutoff method on ion observations in the cusp. The trace points connect the anti-parallel reconnection region with the component reconnection tilted X-line, which is shifted south of the SSP (seasonal effect), and cross over a large part of the magnetopause. These observations indicate the existence of long dayside reconnection lines. The grey line represents the predicted location of the dayside reconnection line using the Maximum Magnetic Shear model (adapted from Fig. 8 in Trattner et al. 2007)

The Polar/TIMAS cusp event observed on 3 June 1996 at 0620 UT with an IMF clock angle of $234^{\circ}$ is shown in Fig. 14. The format is the same as in Fig. 12. The field line trace points from the cusp observations closest to dawn are clustered along the anti-parallel reconnection region of the northern hemisphere dawn sector. Moving towards local noon, the trace points are closely aligned with the component reconnection tilted X-line crossing the dayside magnetopause. This component reconnection location is south of the subsolar point, as expected for an event observed during the northern summer months. The gray line crossing the dayside magnetopause represents the predicted reconnection location from the Maximum Magnetic Shear model, which matches the observed plasma entry location from the cusp observations.

Uncertainties for the Maximum Magnetic Shear model to predict the location of the dayside magnetic reconnection X-line are discussed in Trattner et al. (2007) and include:

1. The size of the dayside magnetosphere being larger/smaller than predicted;
2. Solar wind flow deviations from the Sun-Earth line;
3. IMF diffusion into the model magnetosphere altering the orientation of the field lines.

These individual errors for the location of the reconnection X-line have been estimated as $\leq 1~\text{R}_{\text{E}}$. A subsequent study (Petrinec et al. 2014) investigating the steepness of the magnetopause saddle across the maximum magnetic shear location showed, that the magnetopause magnetic shear within $2~\text{R}_{\text{E}}$ of the predicted location changes by less the $2^{\circ}$, which was adopted as the uncertainty for the model.

With an increasing number of datasets and studies of the dayside magnetic reconnection location, some minor modifications to the Maximum Magnetic Shear model were required and have been implemented. While the original cusp study with 130 events (Trattner et al. 2007) was limited to events observed during southward IMF conditions where the reconnection site is expected to be on the dayside magnetopause, in situ observations at the magnetopause also revealed dayside reconnection locations during northward IMF conditions. An investigation of 33 magnetopause crossings by the Double Star TC1 spacecraft equatorward of the cusps showed accelerated ion beam reversals at the dayside magnetopause and contained seven events observed during northward IMF conditions (Trenchi et al. 2008, 2009).

In a follow-up study, Trattner et al. (2017b) investigated the dayside reconnection locations of the seven events observed during steady northward IMF conditions, finding that these identified dayside reconnection locations also fit the predictions of the Maximum Magnetic Shear model. In addition, one of these TC1 magnetopause crossings occurred during a conjunction with the Polar spacecraft located in the southern cusp region. The cusp observations are used together with the low-velocity cutoff method to independently determine the dayside reconnection region where the cusp ions crossed the magnetopause.

**Fig. 15:**
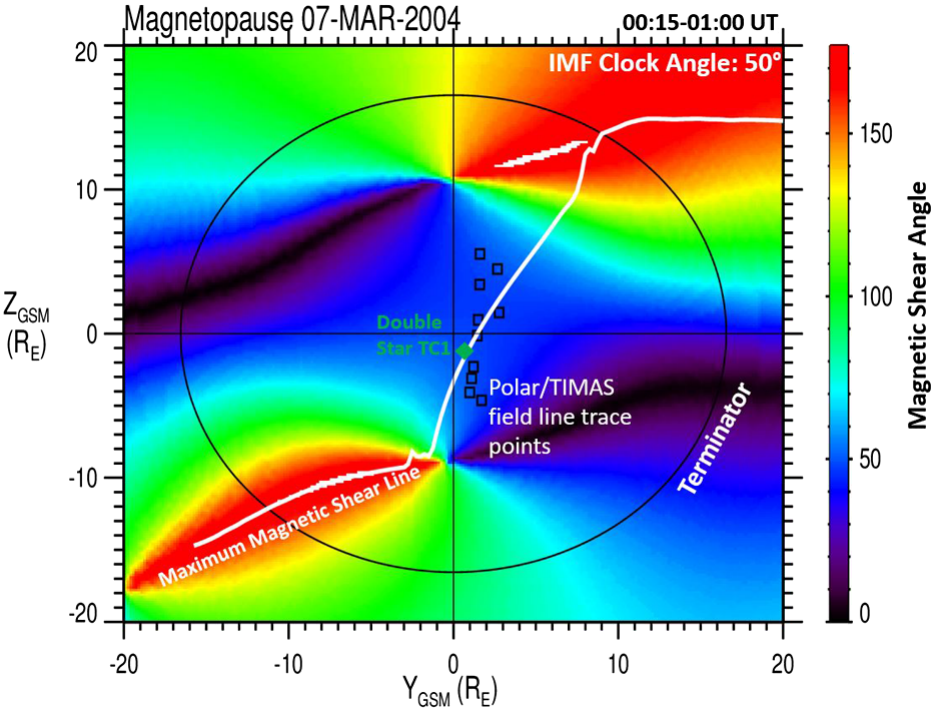
The magnetic shear angle across the magnetopause for 7 March 2004. The green diamond shows the location of the Double Star TC1 satellite at the magnetopause, observing an ion beam switch. The white line crossing the dayside magnetopause is the predicted reconnection location from the Maximum Magnetic Shear model, extended to northward IMF. Black squares represent the field line trace points from a southern hemisphere cusp crossing by Polar (adapted from Fig. 7 in Trattner et al. 2017a, 2017b)

This magnetopause/cusp conjunction event, observed on 7 March 2004 from 00:15 to 01:00 UT with an IMF clock angle of $50^{\circ}$, is shown in Fig. 15. The location of the Double Star TC1 spacecraft, as it crossed the magnetopause and observed the accelerated ion beam switch in the magnetopause boundary layer, is marked by a green diamond. This location almost perfectly matches the reconnection location predicted by the Maximum Magnetic Shear model (white line), while the field line trace points from the Polar/TIMAS cusp observations (black squares) map along the predicted reconnection location.

All three methodologies (direct observations, model predictions, and remote field line tracing) are consistent with the existence of a reconnection location in the subsolar region, as expected from a component reconnection tilted X-line. This study expands the range of use of the Maximum Magnetic Shear model to include cases where dayside reconnection occurs during northward IMF, with the lowest local magnetic shear in this study at about $50^{\circ}$, in agreement with earlier observations by Gosling et al. (1990).

Another modification of the Maximum Magnetic Shear model, based on observations from the MMS mission (e.g., Burch et al. 2016), was implemented. In the original study, the Maximum Magnetic Shear model showed a puzzling anomaly in predicting the dayside reconnection location, which occurs during the fall and spring equinoxes for events with IMF clock angles around $120^{\circ}$ and $240^{\circ}$, respectively. Another previously unknown anomaly was identified in the MMS dataset for events observed around December that also occurs for the same IMF clock angle ranges as the first anomaly.

**Fig. 16:**
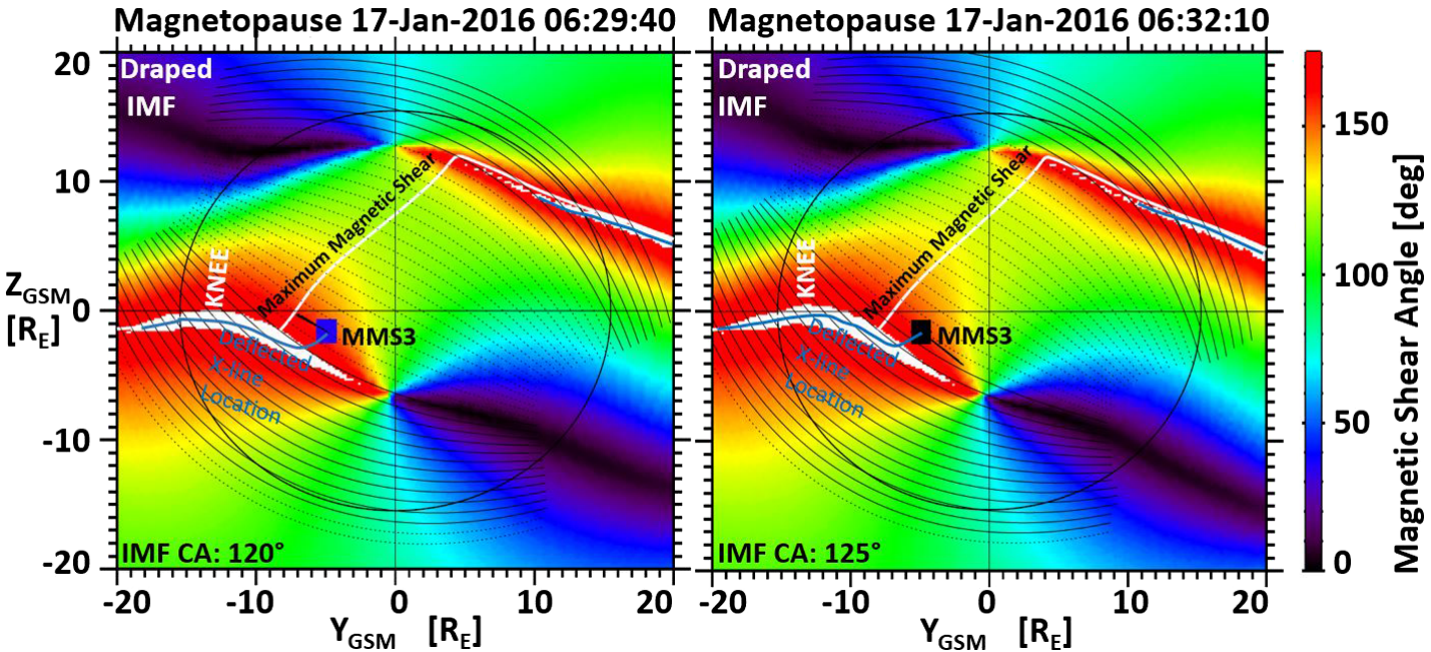
The magnetic shear angle across the magnetopause during an MMS magnetopause crossing on 17 January 2016. IMF draping conditions under large dipole tilts result in the formation of a “Knee” for the dawn anti-parallel reconnection region, which deflects the location of the component reconnection tilted X-line (adapted from Fig. 9 in Trattner et al. 2018)

An example of the second anomaly, known as a “Knee” event, is shown in Fig. 16. The name of the anomaly reflects the unique shape of the anti-parallel reconnection region at the magnetopause, caused by the combination of a large dipole tilt together with the IMF clock angles mentioned above, resembling a bent knee. Shown in Fig. 16 are two magnetic shear angle plots during a MMS3 magnetopause crossing on 17 January 2016 with an IMF clock angle of about $120^{\circ}$. The MMS3 locations in Fig. 16 are marked by blue (MSBL) and black (LLBL) symbols. The black lines emanating from the MMS3 symbols represent the ion beam directions and scaled magnitude in the magnetopause boundary observed by the MMS/FPI instrument.

At 06:29.40 UT (left panel of Fig. 16), the MMS3 spacecraft in the magnetosheath boundary layer was located about $3~\text{R}_{\text{E}}$ south of the X-line predicted by the Maximum Magnetic Shear model and observed a northward ion beam. Such an ion beam can only come from an X-line located south of the spacecraft and disagrees with the model prediction. At 06:32.10 UT (right panel of Fig. 16) the MMS3 spacecraft, now located in the LLBL, observed a southward ion beam in agreement with the predicted reconnection location. This accelerated ion beam switch marks the deflected location of the component reconnection tilted X-line compared to the predicted reconnection location and represents an anomaly for the Maximum Magnetic Shear model.

Overlaid in Fig. 16 are the draped IMF lines in the magnetosheath (thin solid and dashed black lines) that are used to create the magnetic shear angle plots. While the anti-parallel reconnection regions (white area) are usually separated in different hemispheres, the large dipole tilt conditions of this event, together with the IMF clock angle of about $120^{\circ}$, causes the anti-parallel reconnection region in the dawn sector to bend before turning toward the southern cusp, creating the knee-like feature in the anti-parallel reconnection region. That Knee causes part of the anti-parallel reconnection region to line up along the draped IMF direction, which is ultimately responsible for the southward deflection of the component reconnection tilted X-line (Trattner et al. 2018).

The analysis of 11 Knee events observed by MMS showed that the extended dayside X-line follows the knee shape of the anti-parallel reconnection region towards the southern cusp until the draped IMF field lines no longer intercept the anti-parallel reconnection region. That defines the anchor point, connecting the anti-parallel reconnection region to the now new deflected component reconnection region, and coincides in all analyzed cases with the location of the MMS spacecraft and the observed ion beam switches.

These events demonstrate that magnetic reconnection at the Earth’s dayside magnetopause preferentially occurs in the anti-parallel reconnection region and only along component reconnection X-line segments when the draped IMF field lines at the magnetopause no longer have contact with an anti-parallel reconnection region. The new location of the component reconnection tilted X-line in Fig. 16 has not been completed across the magnetopause to the other anti-parallel reconnection region in the dusk sector, since it is currently unclear which of the following possibilities dominates.

From the newly defined deflected anchor point, the X-line could progress northward to the predicted maximum magnetic shear angle location and then match the original configuration of the Maximum Magnetic Shear model. However, it is also possible that the deflected component reconnection tilted X-line simply connects along the shortest path to the duskside, anti-parallel reconnection region, running parallel to and considerably south of the predicted maximum magnetic shear X-line. The broader implications of this result are that anti-parallel reconnection is not only the dominant reconnection scenario, but it also controls the location of the component reconnection line.

## Validating the Maximum Magnetic Shear Model

Since its inception, predictions of the Maximum Magnetic Shear model have been used and validated in several studies involving different methodologies and data sets. One of this methodologies compared various models that predict dayside magnetopause reconnection locations with each other using global resistive MHD simulations (Komar et al. 2015). Models involved in this comparative study are the Maximum Magnetic Shear model (Trattner et al. 2007), maximization of the asymmetric reconnection outflow speed (Swisdak and Drake 2007), maximization of the asymmetric reconnection rate (Borovsky 2013), the angle of bisection (Moore et al. 2002; Borovsky 2008; Hesse et al. 2013), the maximization of the current density magnitude (Alexeev et al. 1998), and component reconnection (Sonnerup 1974; Gonzalez and Mozer 1974). Although the Komar et al. (2015) study compared these prediction models with each other they did not compare models and observations.

Other studies discuss either observations where encounters with magnetopause dayside X-lines are compared with predicted X-line locations from the Maximum Magnetic shear model (e.g., Fuselier et al. 2017; Trattner et al. 2017a,b), or indirect observations where signatures of active magnetopause reconnection, e.g., the directions of accelerated ion beams, are compared with these predicted X-line locations.

**Fig. 17:**
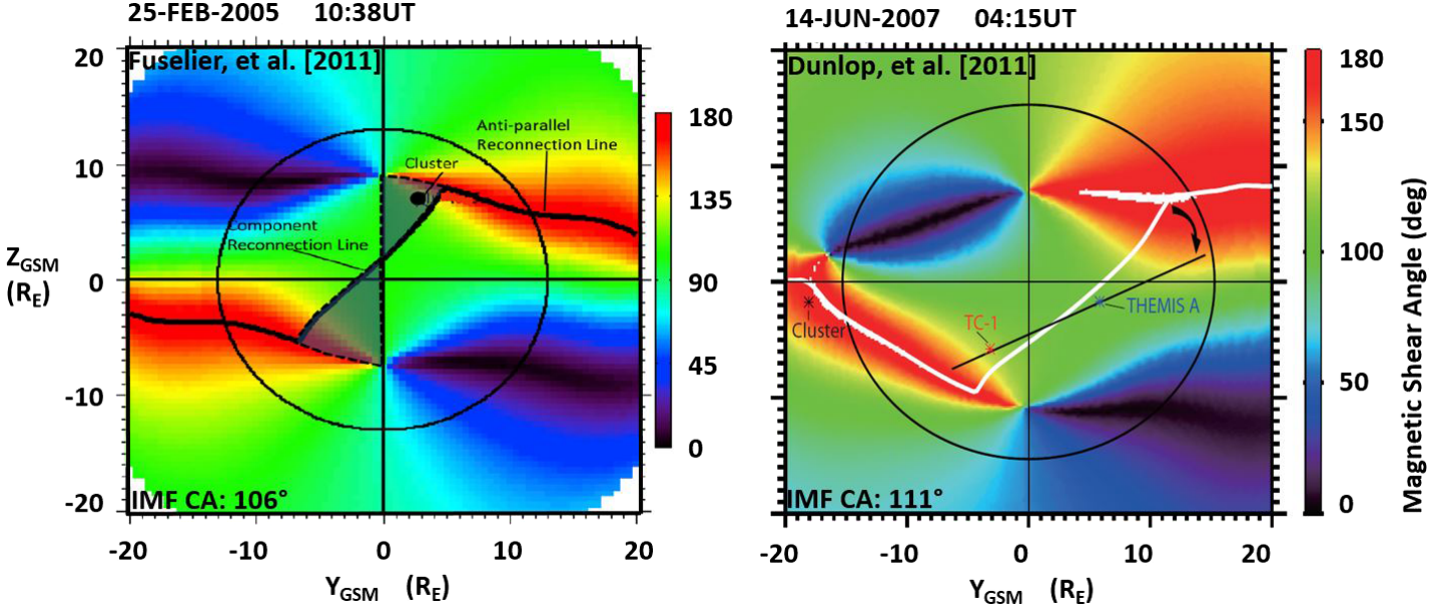
The magnetic shear angle across the magnetopause during a Cluster magnetopause crossing on 25 February 2005 (adapted from Fig. 1 in Fuselier et al. 2011) and a 10-satellite conjunction (Themis, Cluster, Double Star) on 14 June 2007 (adapted from Fig. 3 in Dunlop et al. 2011)

Two examples using the indirect methodology are shown in Fig. 17. The left panel shows a magnetopause magnetic shear angle plot from a study of magnetopause crossings by the Cluster spacecraft (Fuselier et al. 2011). In this example, the Cluster spacecraft crossed the magnetopause on 25 February 2005 at 10:38 UT close to the northern cusp region. This spacecraft location, together with an IMF clock angle of $104^{\circ}$, places the spacecraft between a component reconnection tilted X-line located south of the Cluster location and the anti-parallel reconnection region located in the north (the area is color coded in green in Fig. 17 left panel). The flow directions of accelerated ion and electron beams in the MSBL and LLBL are used to determine the direction to the actual location of the reconnection site with respect to the spacecraft. This particular IMF orientation and spacecraft location provides a tool to distinguish anti-parallel from component reconnection, thus tests the validity of the model predictions.

The study contained a limited number of events, driven by the relatively restrictive conditions whereby this particular test is possible. Of these events, the Maximum Magnetic Shear model appears to accurately predict the correct type of reconnection at the magnetopause in 13 of the 15 events.

The right panel of Fig. 17 shows a magnetopause magnetic shear plot from a multi-spacecraft study by Dunlop et al. (2011). The study uses conjunctions of 10 spacecraft to provide simultaneous monitoring of the dayside magnetopause across a wide range of local times. In the example shown in Fig. 17, the four Cluster spacecraft crossed the dawnside magnetopause at low latitudes, while the five THEMIS spacecraft crossed the low-latitude, dusk-side magnetopause. In addition, the Double star TC-1 spacecraft was in an equatorial orbit between the local times of the THEMIS and Cluster orbits. Accelerated ion beam and FTE signatures observed during the nearly simultaneous magnetopause boundary crossings of these ten spacecraft are consistent with both a tilted X-line at the southern dayside magnetopause and anti-parallel reconnection regions extending along the magnetopause flanks. That is, these observations are consistent with the predictions from the Maximum Magnetic Shear model. The study also demonstrated the occurrence of simultaneous active magnetic reconnection signatures all across the local time sectors covered by the spacecraft.

The predictions of the Maximum Magnetic Shear model were also used in a study of neutral atom emissions from the dayside magnetosheath, LLBL, and cusp observed remotely by the Interstellar Boundary Explorer (IBEX) (Petrinec et al. 2011). Energetic ions from the dayside reconnection site enter the magnetospheric cusps, where a fraction of the ions interact and charge exchange with neutral hydrogen of the geocorona. The resulting energetic neutral atoms (ENAs) propagate away from the dayside regions and are observed by IBEX.

**Fig. 18:**
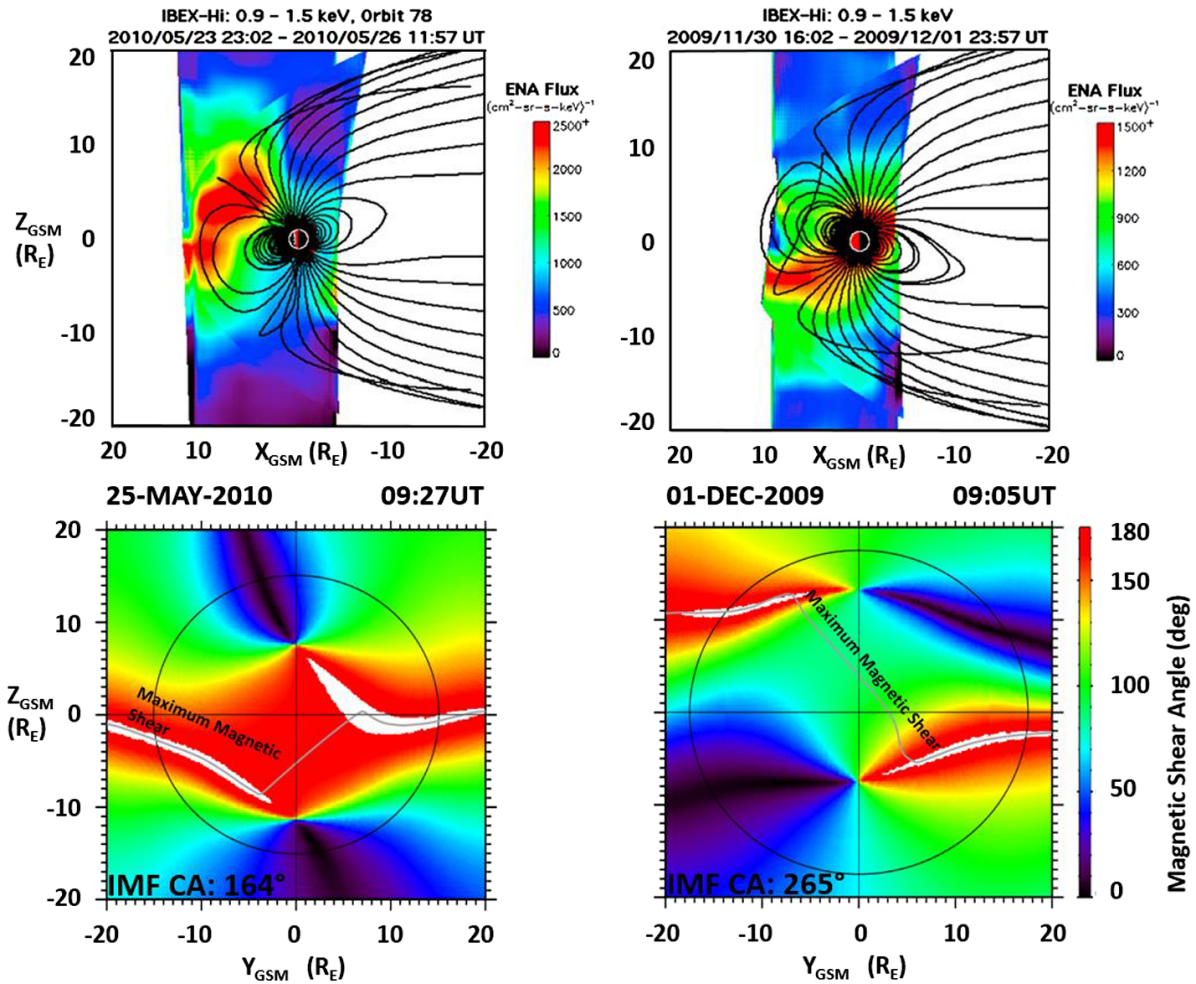
Composite images of the magnetospheric cusps observed by IBEX with the associated magnetic shear angle plots for the average solar wind and IMF conditions during the two IBEX observations (adapted from Fig. 5 in Petrinec et al. 2011)

As shown in Fig. 18 (top panels), the ENA cusp emissions have a strong asymmetry between the northern and southern cusps, and that asymmetry is a function of the Earth’s dipole tilt angle. The observations shown in the panels only include times for which the IMF was southward and occurred during different seasons. In both cases the summer hemisphere cusp region exhibited significantly brighter ENA emissions than the winter hemisphere.

The bottom panels of Fig. 18 show the magnetic shear angle across the magnetopause for the midpoint of the ENA observations time interval above, using the average solar wind and IMF conditions for the observation time interval. The grey lines illustrate where magnetic reconnection is predicted to occur across the dayside magnetopause (left panels), using the Maximum Magnetic Shear model. As expected for a positive dipole tilt angle, most of the component reconnection tilted X-line lies below the magnetic equator. Flux tubes reconnecting at this southern hemisphere X-line and attached to the southern cusp quickly convect to the nightside, with low magnetosheath number flux. In contrast, flux tubes reconnecting at this X-line and attached to the northern cusp convect slowly since they must first move against the ambient magnetosheath flow until reaching the geomagnetic equator. This allows for longer convection times and a much greater magnetosheath number flux to enter the northern cusp and interact with the geocorona, causing more intense ENA emissions. The reverse is true during the winter months and negative dipole tilt, where the southern cusp appears brighter in ENAs than the northern cusp (right panels in Fig. 18).

The Maximum Magnetic Shear model was used extensively during the MMS mission development phase to target the magnetopause reconnection location, creating probability maps where magnetopause reconnection would most likely occur, using solar wind input conditions observed during the previous solar cycle (Griffiths et al. 2011). This study was also used to predict how many encounters with the reconnection region MMS could expect during the prime mission (Fuselier et al. 2016).

The launch of the MMS spacecraft thus provided new possibilities to validate the predictions from the Maximum Magnetic Shear model. The first phases of the prime mission, designated as phases 1a and 1b, were designed to skim the magnetopause and maximize encounters with the dayside reconnection line across the entire dayside. Using the above-described methodology of identifying accelerated ion beams in the magnetopause boundary layers that switch flow direction during the MMS crossing, 302 X-line encounters were identified during phase 1a of the MMS mission (Trattner et al. 2017a). These observed locations of the dayside magnetopause X-line were compared with predictions of the Maximum Magnetic Shear model. The results of the comparison are summarized in Table 1. Of 302 events, 82% (249 events) are in agreement and within the defined error bar of $\pm2~\text{R}_{\text{E}}$ around the predicted X-line (Petrinec et al. 2014). This percentage agreement is about 2% higher than that reported in the original study (Trattner et al. 2017a, 2017b). The difference is due to the recent published solution for events known as the “Knee” anomaly (see above).

**Table 1:**
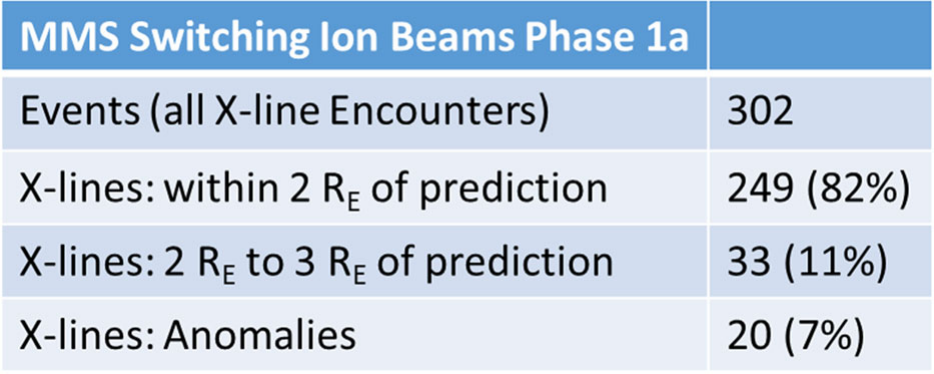
The distance to the predicted location of the magnetopause X-line (Maximum Magnetic Shear model) for 302 MMS X-line locations. 82% of the observed X-lines are within $2~\text{R}_{\text{E}}$ of the predicted location and within the uncertainties for the model. Prediction anomalies occur during the equinoxes for IMF clock angles around $240^{\circ}$ and $120^{\circ}$ (Trattner et al. 2017a)

Magnetopause X-lines located between $2~\text{R}_{\text{E}}$ to $3~\text{R}_{\text{E}}$ around the predicted X-line account for 11% of the survey (33 events). These events are most likely caused by changing solar wind and IMF conditions during the MMS magnetopause encounters and the result of the uncertainty in the magnetopause response time for the reconnection location.

The study also confirmed the existence of events with larger distances between the observed and predicted reconnection locations. These events are listed as anomalies in Table 1 and represent 7%, or 20 events, of the X-line survey. The anomaly events cluster around the spring and fall equinoxes at IMF clock angles around $240^{\circ}$ and $120^{\circ}$, respectively. This unsolved anomaly has also been documented in earlier reconnection location studies, including the original cusp study using the low-velocity cutoff methodology to determine the reconnection site (Trattner et al. 2007), as well as direct observations of X-lines at the magnetopause by the Double Star TC1 probe (Trattner et al. 2017b). The proximity of the anomaly to the equinoxes suggests a symmetry effect where an X-line location that is close to the subsolar region, together with a symmetric plasma flow around the magnetopause, seems to become unstable and jumps to either the northern or southern hemisphere.

**Fig. 19:**
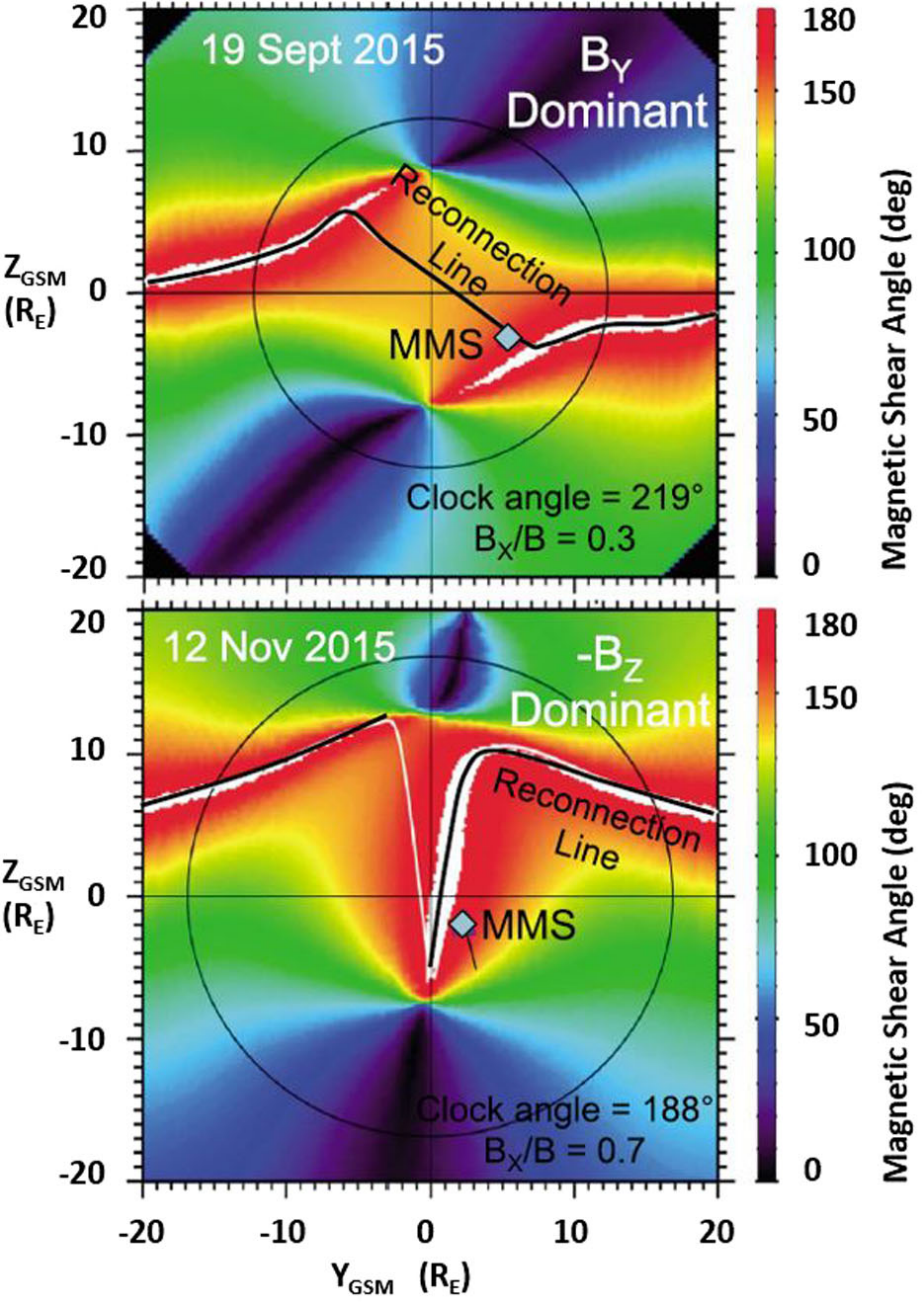
The magnetic shear across the magnetopause for two MMS magnetopause crossings, where an electron diffusion region (EDR) was identified. The reconnection locations are compared with predictions from the Maximum Magnetic Shear model (adapted from Fig. 2 in Fuselier et al. 2017)

The first 12 EDR encounters identified in MMS mission phase 1a were investigated by Fuselier et al. (2017) for their agreement with the predicted location from the Maximum Magnetic Shear model, the stability of the reconnection X-line location, and the presence of other X-lines in the vicinity of the events. EDR encounters are particularly useful for studying predicted X-line locations because the EDR locates the X-line within a few 10s of km. Two of these EDR events are shown in Fig. 19. In the magnetopause magnetic shear plots, black lines indicate the predicted locations of the X-lines. The black circles show the location of the terminator plane, and the blue diamonds show the location of the MMS spacecraft at the EDR encounters.

The top panel in Fig. 19 shows the EDR event observed on 19 September 2015. The IMF clock angle for this event was $219^{\circ}$, and this event represents an IMF B_Y_ dominant X-line event, which turned out to be the most common category for all identified EDRs. For IMF B_Y_ dominant conditions, the Maximum Magnetic Shear Model predicts that a continuous antiparallel and component reconnection line extends across the entire dayside magnetopause (Trattner et al. 2007), with anti-parallel reconnection on the flanks of the magnetopause connected by the component reconnection line. The MMS spacecraft are located in the dusk southern hemisphere very close to the predicted, component reconnection X-line.

The bottom panel of Fig. 19 shows the magnetopause magnetic shear conditions during the 12 November 2015 EDR event. The IMF clock angle for this event was $188^{\circ}$, and this event represents an IMF B_Z_ dominant X-line. For these conditions, the Maximum Magnetic Shear Model predicts a pair of anti-parallel reconnection lines extending from the magnetospheric cusps towards the flanks (Trattner et al. 2007). The shape of these anti-parallel reconnection lines in Fig. 19 (bottom panel) is highly distorted because of the relatively large value of the IMF B_X_ component during the magnetopause crossing. However, for this EDR encounter the MMS spacecraft are again relatively close to the anti-parallel reconnection line (within $2~\text{R}_{\text{E}}$).

All but four EDR events in the phase 1a survey are observed within the $2~\text{R}_{\text{E}}$ uncertainty of the Maximum Magnetic Shear model. Of the four EDR events, which are more than $2~\text{R}_{\text{E}}$ distance to the predicted X-line, one is associated with strongly northward IMF and a Kelvin-Helmholtz (KH) wave event. Thus, this event is technically outside of the IMF limits of the Maximum Magnetic Shear model. The other three events occurred when the IMF B_X_ component was very large ($|\text{B}_{\text{X}}|/\text{B} > 0.8$). Under these radial IMF conditions, the model used to determine the draping of the magnetosheath field lines is considered inaccurate (Trattner et al. 2007, 2012a), and the increased distance is not considered a failure of the model but requires more study. Thus, for the applicable parameter range of the other eight X-line events, the Maximum Magnetic Shear model does a reasonably good job of predicting the location of the X-line in the vicinity of the spacecraft.

A recent innovative methodology to determine the distance along the field line between the dayside magnetopause reconnection location and an observer was developed by Broll et al. (2017). The method is designed for southward IMF conditions and uses only ion-velocity distribution measurements from a single spacecraft to determine the distance. This distance is subsequently compared to the reconnection location predicted by the Maximum Magnetic Shear model

The reconnection exhaust for ions injected at the dayside reconnection location is predicted to be a characteristic D-shape distribution (Cowley 1982). For this reason, the deformation of the magnetosheath distribution in the vicinity of the cutoff velocity reflects the change in magnetic field strength experienced by the ions on their way to the cusp regions, thus, the distance the observed D-shaped distribution traveled between the reconnection X-line and the observation point.

**Fig. 20:**
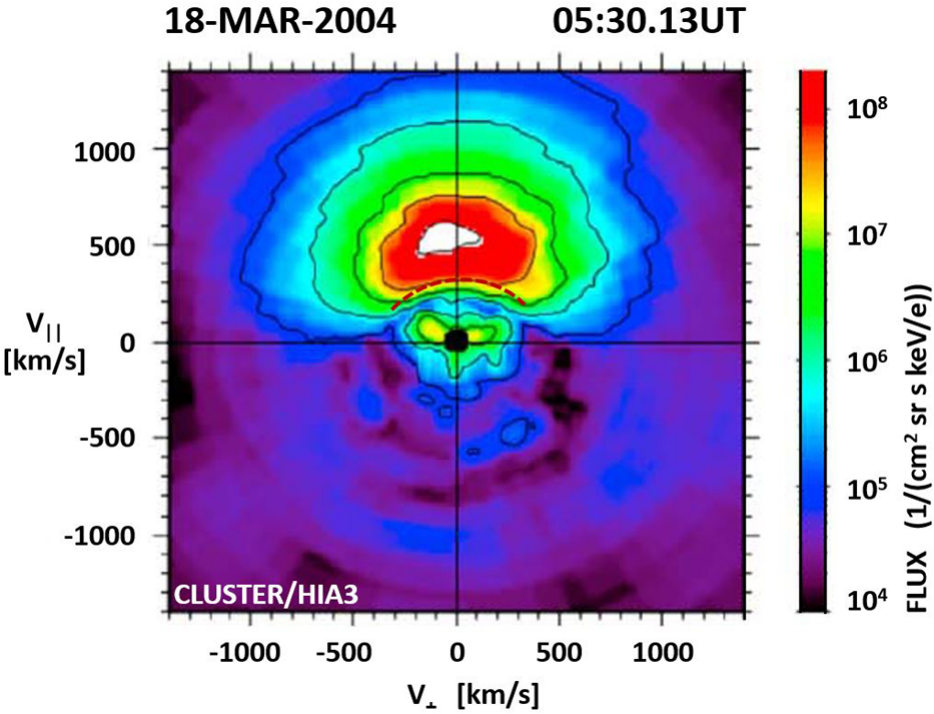
Magnetopause boundary layer ion distribution observed by Cluster/HIA3 on 18 March 2004. The curvature (dashed line) of the evolved D-shape distributions is used to determine the distance to the X-line (adapted from Fig. 4 in Broll et al. 2017)

Figure 20 shows the magnetopause boundary-layer distribution observed by Cluster/HIA3 on 18 March 2004. The deformation or curvature of the evolved D-shaped distribution that is used to determine the distance to the reconnection site is identified with a dashed black line. The methodology involves a simulation where ions are propagated through a model magnetosphere to determine how the D-shaped distribution evolves away from the reconnection X-line. For the model magnetosphere, the study uses the Tsyganenko semi-empirical model (e.g., Tsyganenko and Sitnov 2005). Ion distributions are produced at $0.1~\text{R}_{\text{E}}$ increments along the field line for comparison with the observed distributions. The results of the events tested in this study are in agreement with the predicted locations of the Maximum Magnetic Shear Model.

Another methodology to validate predictions form the Maximum Magnetic Shear model was introduced by Fuselier et al. (2019). The event study discussed two MMS magnetopause crossings during large dipole tilt conditions, when the predicted X-line is shifted significantly away from the subsolar region. With MMS located in the magnetopause boundary layer in the subsolar region, the spacecraft are far enough away from the predicted X-line that they observe both the accelerated ion beam from the X-line and also the return beam mirrored from the ionosphere, before the newly opened magnetic field line convects over the spacecraft and cuts the return beam off. With both beams present, the low-velocity cutoff method described above is used to determine the distance to the reconnection site. Following the procedure for cusp events, the calculated distance to the reconnection site was traced back along the magnetic field direction and agreed with the predicted X-line location within the uncertainty of the distance calculation.

In contrast to an, e.g. MHD model, the Maximum Magnetic Shear model is an empirical model based on cusp observation where the time of flight characteristic of precipitating and mirrored magnetosheath ions are used to remotely sense the location of the magnetopause reconnection X-line. The predicted locations of reconnection X-lines at the magnetopause from this model have been verified using an array of tests and spacecraft data sets. In addition, this model accurately predicted the number of MMS magnetopause crossings within $\sim0.5~\text{R}_{\text{E}}$ of a reconnection diffusion region (Petrinec et al. 2016) and the $\sim32$ in situ reconnection EDR encounters during the prime mission (Webster et al. 2018). This serves as a testimony to successful mission planning using this model. The number and diversity of these tests make the Maximum Magnetic Shear model one of the more successful models for the location of reconnection over the entire dayside magnetopause, spanning a wide range of IMF orientations.

## Variability of Magnetic Reconnection

### Flux Transfer Events

Magnetic reconnection at the magnetopause can be transient, and can occur over relatively small ($\sim1~\text{R}_{\text{E}}$ or smaller) spatial scales. Such intervals of sporadic ($\sim1\text{--}2$ minute), small-scale magnetic reconnection are known as FTEs, as they were envisioned to transfer particle and energy flux from the shocked solar wind into the magnetosphere via this process (Russell and Elphic 1978, 1979). The classic observational signature of an FTE at the magnetopause generally includes a transient increase in the magnetic field intensity coincident with a bipolar signature along the normal to the magnetopause surface (although “crater” FTEs have also been described Farrugia et al. 2011). Observations associated with FTEs include a draped magnetic field region and a central “core” region that consists of twisted, reconnected flux tube(s) containing both magnetosheath and magnetospheric plasmas (Rijnbeek et al. 1987).

Many subsequent studies have used plasma and field observations to examine the topologies and characteristics of FTEs in detail (e.g., Paschmann et al. 1982; Klumpar et al. 1990; Owen et al. 2001; Pu et al. 2013; Roux et al. 2015; Fuselier et al. 2017; Petrinec et al. 2020), including the complex processes occurring between interacting FTEs (e.g., Øieroset et al. 2011, 2016; Fargette et al. 2020; Russell and Qi 2020). In the context of this review, the focus herein is on the occurrence of FTEs: both in isolation and in relation to the large-scale expected reconnection line location.

FTEs have been observed by various spacecraft to occur often in the vicinity of the magnetopause, with a mean separation time of $\sim8~\text{min}$ (Russell et al. 1996). While some have reported FTE signatures to be equally prevalent in the magnetosheath and within the magnetosphere (Kawano and Russell 1996); others have noted a marked preference for FTE observations in the magnetosheath relative to the magnetosphere (Rijnbeek et al. 1984; Kuo et al. 1995; Neudegg et al. 2000; Wang et al. 2005).

FTEs at the dayside magnetopause were found to occur preferentially during periods of southward IMF (e.g., Berchem and Russell 1984; Kawano and Russell 1997; Wang et al. 2005, 2006; Fear et al. 2009, 2010; Eastwood et al. 2012). However, no clear preference for IMF polarity was observed on the magnetopause flanks (Kawano and Russell 1997). This may be due to the motion of dayside magnetopause FTEs being convected to high latitudes during strongly southward IMF conditions, thus not being observed as often at the lower-latitude magnetopause flanks. Dayside magnetopause FTE occurrence has generally been found to be consistent with component reconnection lines that are tilted as a consequence of the IMF clock angle (Rijnbeek et al. 1984; Daly et al. 1984), leading to the conjecture that FTEs may originate from component reconnection lines (Russell et al. 1985). This hypothesis has been supported by numerical simulations (e.g., Raeder 2006; Hoilijoki et al. 2017) and analytic modeling (Sibeck 2009). However, further observational investigation of the relation between FTE origination and extended reconnection lines at the dayside magnetopause is needed. FTE signatures have also been described at the magnetopauses of other planets, as described in Sect. 8 of this paper.

### Simultaneous Multiple X-Lines at the Magnetopause

In addition to the mounting evidence for single, long X-lines across the dayside magnetopause (e.g., Fuselier et al. 2002; Phan et al. 2000; Trattner et al. 2007; Dunlop et al. 2011), observations and simulations also showed evidence for the simultaneous occurrence of multiple X-lines covering a wide range of scale sizes (e.g., Lee and Fu 1985; Drake et al. 2006b; Vines et al. 2017).

Confined close to the EDR, simulations predict the formation of magnetic islands or helical magnetic structures with a scale size of about 100 km on either side of the X-line (e.g., Drake et al. 2006b; Daughton et al. 2011; Nakamura et al. 2016; Eastwood et al. 2016), which may play an important role in the plasma energization (e.g., Drake et al. 2006a). Parallel X-lines separated at intermediate scales of $\sim1~\text{R}_{\text{E}}$ across the magnetopause often show very complex internal structures related to FTEs (e.g., Kacem et al. 2018; Øieroset et al. 2016) (see Sect. 6) and have also been described as flux ropes in three dimensions (e.g., Lee and Fu 1985).

However, there is also evidence of simultaneous multiple X-lines at the magnetopause that are separated by many $\text{R}_{\text{E}}$ (e.g., Fuselier et al. 2011, 2018; Hasegawa et al. 2010; Trattner et al. 2012b; Vines et al. 2017), which would encompass a large portion of the dayside magnetopause. Such large-scale distances between multiple X-lines are also seen in global simulations of the magnetopause (e.g., Chen et al. 2017; Hoilijoki et al. 2017; Raeder 2006).

**Fig. 21:**
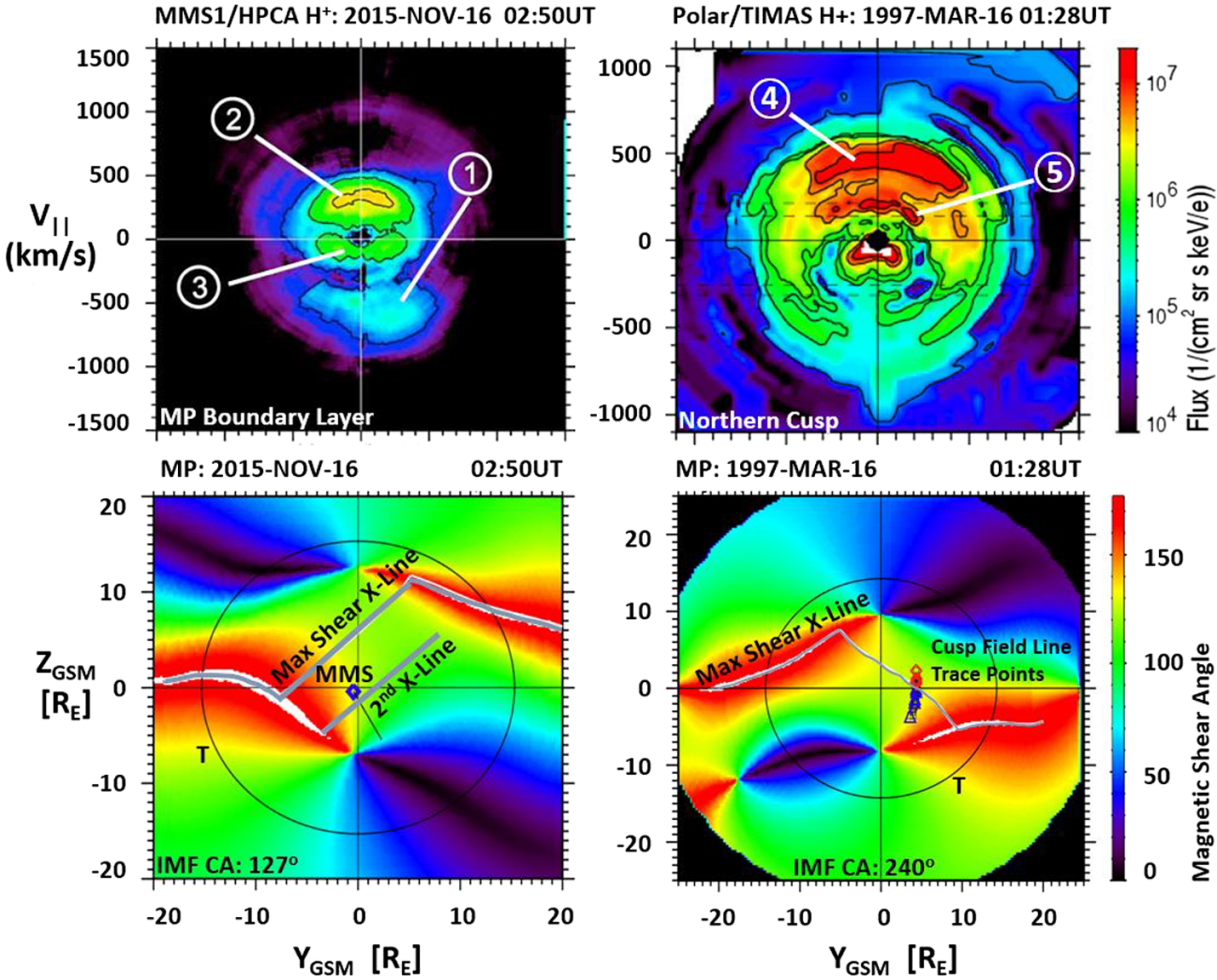
Overlapping $\text{H}^{+}$ distributions in the magnetopause boundary layer and the cusp region (top panels) and their associated magnetic shear angle plots, showing the locations of multiple X-lines at the magnetopause (bottom panels). The panels are adapted from Figs. 6 and 8 in Fuselier et al. (2018) (left) and from Figs. 11 and 12 in Trattner et al. (2012b) (right)

Two examples of multiple magnetopause X-lines that are separated by large distances are shown in Fig. 21. The left panels show an event observed by MMS1 on 16 November 2015 at 02:50 UT (Fuselier et al. 2018) as the spacecraft crosses the magnetopause boundary layer. The top left panel shows three proton distributions (Flux [$1/(\text{cm}^{2}\,\text{sec}\,\text{sr}\,\text{keV/e})$]), observed by the HPCA instrument on MMS1 and plotted in field-aligned coordinates with the perpendicular bulk velocity removed.

Population 1 propagates anti-parallel to the magnetic field and originates at the main X-line located north of the spacecraft. Throughout the MMS1 boundary-layer interval, the peak flux of population 1 decreases progressively while the magnitude of its bulk velocity increases simultaneously (not shown). At the end of the boundary-layer interval, the peak flux of population 1 abruptly increases back to near the original intensity observed at the beginning of the interval when the spacecraft was only connected to the main northern X-line.

While population 1 experiences these changes in peak flux and velocity, population 2, propagating parallel to the magnetic field, does the opposite. The peak flux of population 2 steadily increases throughout the boundary layer interval until it is the dominant distribution then abruptly decreases at the end of the interval. In addition, the bulk velocity of population 2 steadily decreases towards the end of the interval. Population 2 originates at a secondary, possibly transient, X-line south of the spacecraft location. Population 3 is identified as a magnetospheric ion population, which was confirmed using composition measurements by the HPCA instrument. This distribution has a small but variable parallel velocity.

The left bottom panel of Fig. 21 shows the magnetopause magnetic shear plot for the 16 November 2015 boundary-layer crossing by the MMS spacecraft. The format is the same as in Fig. 12. The primary component reconnection tilted X-line, as predicted by the Maximum Magnetic Shear model, is located north of the MMS location at the subsolar region (marked with a blue symbol). On the flanks, the predicted X-line follows the high-shear, anti-parallel reconnection region. The primary component reconnection X-line is the entry location for population 1 as shown in the top left panel. The secondary X-line is located near the subsolar point. The locations for the primary and secondary X-lines across the dayside magnetopause are quasi-stationary.

During the 1.5-min boundary-layer crossing, when the ion beams from the primary/secondary X-lines are observed, the low-velocity cutoff of the parallel propagating population 2, emanating from the secondary X-line south of the spacecraft location, is a straight line. This straight cutoff indicates that these protons crossed the magnetopause at an X-line that was located near the spacecraft. In contrast, the low-velocity cutoff of the anti-parallel propagating population 1 from the primary X-line north of the spacecraft is curved, indicating that these protons crossed at an X-line that was far from the spacecraft.

The fact that the low-speed cutoff of population 1 from the primary X-line remains curved throughout the entire boundary-layer crossing indicates that the primary X-line did not move (Fuselier et al. 2018). This stationary behavior stands in contrast to observations of FTEs and to some simulations of multiple X-lines (e.g., Hoilijoki et al. 2017; Raeder 2006). An FTE propagates along the magnetopause with the solar wind convection, and, if the FTE is bound by two X-lines, then the two X-lines also propagate along the boundary.

The formation of a second entry point at the magnetopause reopens an already open and convecting flux tube. In global MHD simulations, such multiple magnetopause X-lines would occur preferentially for large dipole tilt conditions, while small dipole tilt conditions during equinox are dominated by single X-line reconnection (Raeder 2006, 2009). The occurrence of multiple magnetopause X-lines also leaves a distinctive signature in the precipitating ion distributions observed in the cusps. These cusp signatures appear as overlapping precipitating ion beams at different energies, especially for ions with zero-degree pitch angles (e.g., Onsager 1994; Xue et al. 1997; Fuselier et al. 1997; Trattner et al. 1998).

An event discussing overlapping precipitating ions in the cusp and the associated magnetopause entry locations is shown in Fig. 21 (right panels). The event was observed by Polar/TIMAS on 16 March 1997 at 01:28 UT (Trattner et al. 2012b). The top right panel in Fig. 21 shows a 2D cut of the proton ion flux distribution for the 16 March 1997 Polar cusp crossing, again in field-aligned coordinates with the perpendicular bulk velocity removed. The Polar/TIMAS observation for this time frame shows two clearly separated ion distributions (populations 4 and 5) streaming parallel to the ambient magnetic field, indicating the formation of a second plasma entry point at the magnetopause. The low-velocity cutoff methodology described above is used on both ion distributions to determine their respective distances to the magnetopause X-lines.

The magnetopause magnetic shear angle plot for the Polar cusp crossing on 16 March 1997 at 01:28 UT is shown in the bottom right panel of Fig. 21. Since the event occurred close to equinox, the line of maximum magnetic shear (gray line) crosses the dayside magnetopause close to the subsolar region (e.g., Trattner et al. 2007). The T96 field line trace points for both cusp distributions are located in the equatorial region in the component reconnection region around the predicted line of maximum magnetic shear. The trace points are spread out over several $\text{R}_{\text{E}}$ perpendicular to the line of maximum magnetic shear (grey), and marked with blue triangles for the original cusp ion dispersion and red diamonds for the overlapping ion distribution.

The trace points for the original cusp ion dispersion are located south of the maximum magnetic shear reconnection line, while the trace points for the overlapping distribution are located north of the reconnection line. These locations depict the formation of a magnetic island or flux rope with the size of the magnetic island or distance between the multiple X-lines determined to be between 2 and $5~\text{R}_{\text{E}}$. This scenario is consistent with the multiple X-line model by Lee and Fu (1985) and closely resembles the results of hybrid simulations by Omidi and Sibeck (2007).

### Continuous Versus Intermittent Reconnection

In addition to determining the location of magnetic reconnection at the dayside magnetopause, a persistent problem is understanding whether the reconnection process is primarily continuous or intermittent, or what input conditions might favor one type of reconnection over the other. As discussed above, continuous or steady-state magnetic reconnection at the magnetopause causes a smooth and continuous latitudinal dispersion profile for precipitating magnetosheath ions in the cusps (e.g., Onsager et al. 1993; Lockwood 1997). This type of cusp ion energy dispersion is observed but is also not very common.

The most prominent example for continuous reconnection at the magnetopause is a reconnection event observed by the Cluster and IMAGE spacecraft during sustained northward IMF conditions. The Cluster spacecraft, located in the northern polar region on an outward trajectory across the magnetopause boundary layer, observed a reversal of the accelerated ion beams from the X-line. Simultaneously, the IMAGE spacecraft observed a continuous bright spot poleward of the auroral oval in the ionosphere caused by the precipitating magnetosheath ions (e.g., Frey et al. 2003a,b; Phan et al. 2003). The location of the ionospheric spot was magnetically connected to the location of the Cluster spacecraft at the magnetopause reconnection location. The ionospheric spot was observed for several hours, which was interpreted as evidence for a continuous reconnection process.

Cusp ion energy dispersions caused by precipitating magnetosheath ions are used to determine the distance to the reconnection site (Eq. (1)), and the “Time since Reconnection” occurred at the magnetopause, $\Delta t$, for the now open geomagnetic field line (Eq. (2)). With $t_{\text{SAT}}$ representing the time at the observing spacecraft, the cusp profile of the resulting reconnection time $t_{\text{REC}} = t_{\text{SAT}} - \Delta t$, either continuous or stepped, is a direct measurement of the nature of magnetic reconnection at the reconnection site (Trattner et al. 2015).

**Fig. 22:**
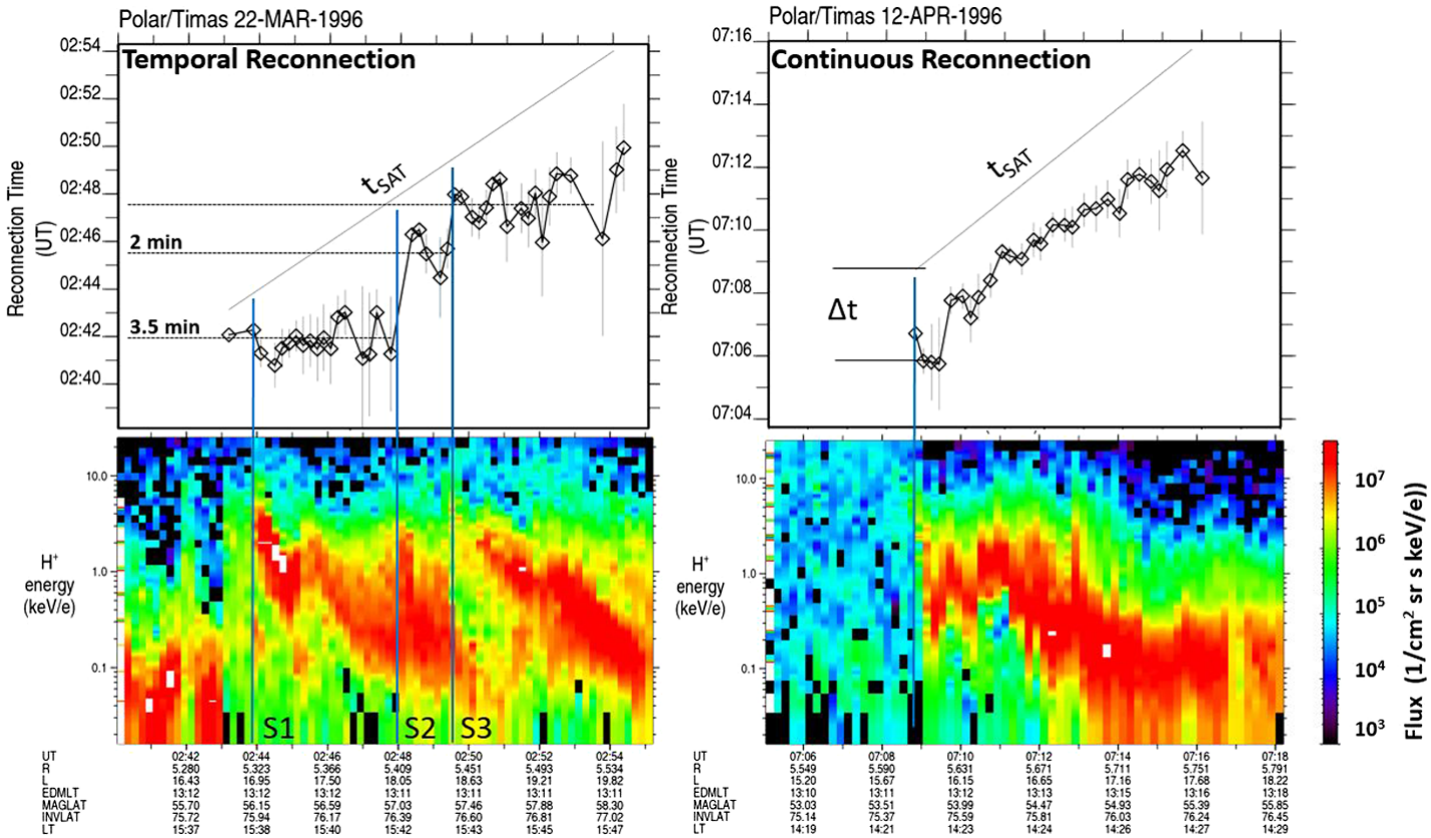
Stepped $\text{H}^{+}$ ion distributions in the northern cusp, observed by Polar/TIMAS on 22 March 1996 (bottom left panel) and 12 April 1996 (bottom right panel) (adapted from Figs. 5 and 9 in Trattner et al. 2015). Using a modification to the low-velocity cutoff methodology, the reconnection time can be calculated, which is either stepped or continuous for temporal/pulsed and continuous reconnection events, respectively

Two examples for temporal and continuous reconnection using the “Time since Reconnection” methodology are shown in Fig. 22. The bottom left panel of Fig. 22 shows flux measurements for downward precipitating magnetosheath ions in the pitch angle range from $0^{\circ}$ to $15^{\circ}$ during the 22 March 1996 Polar/TIMAS cusp crossing. The panel shows three step-up cusp structures, S1 to S3, within the 02:40 to 02:55 UT time range, marked with vertical blue lines.

The top left panel in Fig. 22 shows $t_{\text{REC}}$, the time when the open field lines in the cusp encountered by the Polar spacecraft were reconnected at the magnetopause reconnection location. The first major cusp structure (S1) observed during the 22 March 1996 Polar/TIMAS cusp crossing was encountered by the Polar spacecraft at 02:44 UT. The top left panel in Fig. 22 reveals that the magnetic field lines associated with cusp step S1 were reconnected at the magnetopause 2 min earlier at 02:42 UT. In addition, the profile of $t_{\text{REC}}$ across cusp structure S1 reveals that the structure was caused by one brief reconnection pulse after which the magnetopause reconnection process dramatically decreased or ceased altogether.

The following step-up cusp structure S2 was encountered by Polar/TIMAS at 02:48 UT. For this cusp structure, the average $t_{\text{REC}}$ was calculated at 02:45:30 UT, 3.5 min after the first reconnection pulse S1. The final cusp structure S3 was encountered by Polar/TIMAS at 02:49:30 UT with a $t_{\text{REC}}$ calculated at 02:47:30 UT, 2 min after reconnection pulse S2.

All magnetic field lines for the individual cusp structures S1–S3 encountered during the 22 March 1996 cusp crossing show a $t_{\text{REC}}$ that is approximately constant during the crossings of the structures. The constant $t_{\text{REC}}$ indicates that this event is a pulsed reconnection event.

The right panels of Fig. 22 show a Polar spacecraft crossing of the cusp on 12 April 1996. Polar encountered downward precipitating magnetosheath ions at about 07:09 UT and observed a single ion energy dispersion marked with a vertical blue line. The top right panel in Fig. 22 shows the $t_{\text{REC}}$ for the Polar cusp crossing and reveals that the cusp magnetic field lines at the open-closed field line boundary were reconnected at the magnetopause 3 min earlier at 07:06 UT. However, throughout that cusp crossing, $t_{\text{REC}}$ rises continuously at approximately the same rate as the line representing the time at the observing spacecraft (thin black line in the top right panel of Fig. 22), indicating that magnetic reconnection at the magnetopause was a continuous process.

While observational evidence for both types of reconnection (continuous and pulsed) exists, the questions of which type dominates at the magnetopause or which internal or external conditions are responsible for one type or the other are still unresolved.

### Magnetic Reconnection Memory

Since the Maximum Magnetic Shear model only considers the magnetic shear across the dayside magnetopause to determine the location of the X-line, it responds instantaneously to small and large changes in solar wind/IMF conditions. However, in nature a delay time is expected between the rotation of the IMF and when the new location of the magnetopause X-line associated with the rotated IMF is established (Trattner et al. 2016).

**Fig. 23:**
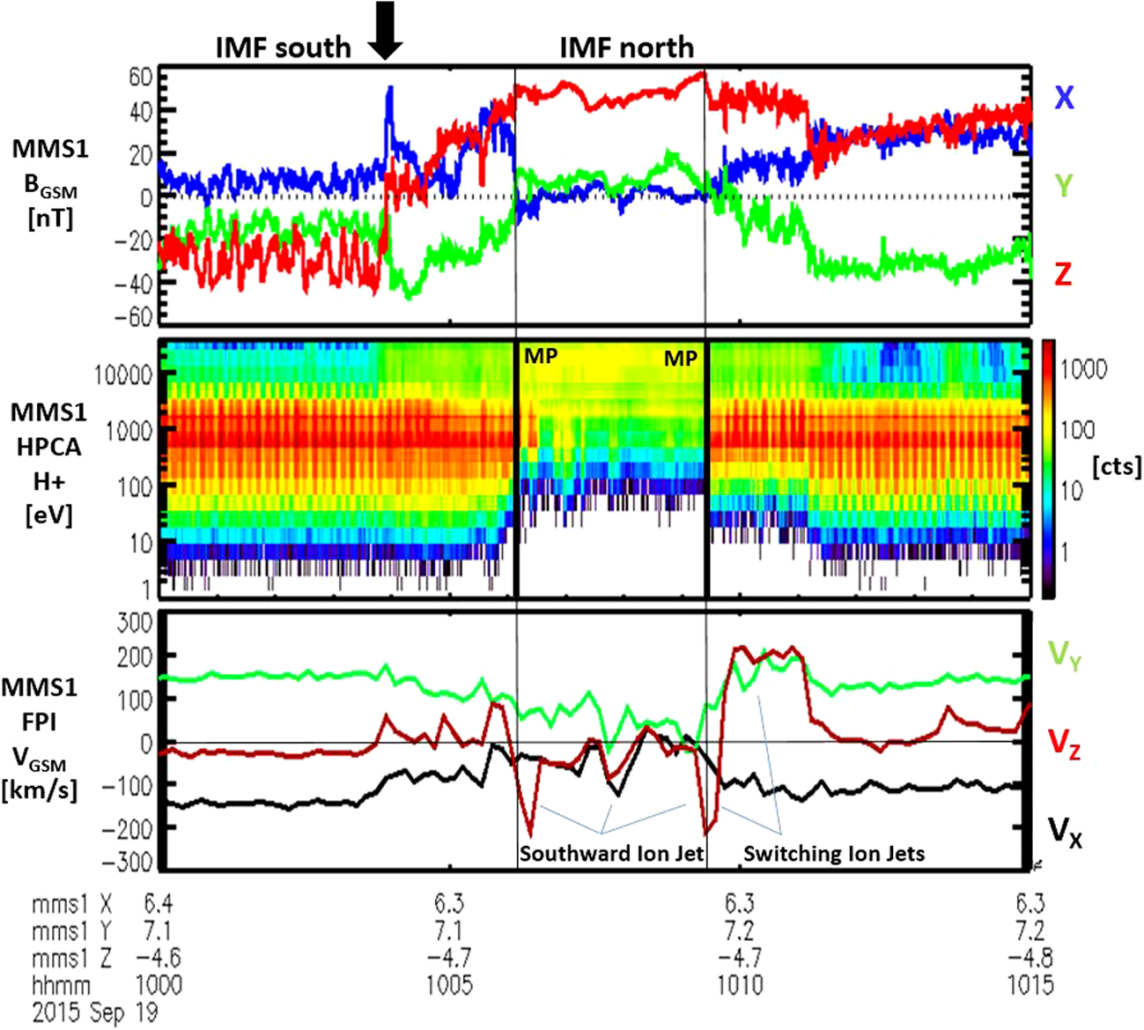
The MMS magnetopause encounter on 19 September 2015. Shown from top to bottom are, the MMS1/FGM magnetic field components (GSM), the MMS1/HPCA proton energy-time spectrogram (cts), and the MMS1/FPI plasma velocity moments (km/s) (adapted from Fig. 4 in Trattner et al. 2016)

Figure 23 shows an MMS magnetopause encounter on 19 September 2015 where the spacecraft crossed the magnetopause twice at about 10:06 and 10:09.30 UT. Plotted from top to bottom are the MMS1/FGM magnetic field components in GSM coordinates, the MMS1/HPCA proton energy-time spectrogram [cts], and the MMS1/FPI plasma velocity moments [km/s]. The MMS spacecraft approached the magnetopause from the magnetosheath and were located close to and slightly south of the predicted location of the magnetopause X-line (not shown). At 10:04 UT, MMS observed a sudden sharp northward rotation of the IMF, which changed the IMF clock angle from $258^{\circ}$ to $316^{\circ}$. The IMF remained northward for the rest of the event including the two magnetopause crossings.

The MMS spacecraft crossed the magnetopause boundary layer and encountered geomagnetic field lines at about 10:06 UT. During the boundary-layer crossing, MMS observed a southward ion jet, which shows that the reconnection X-line was still north of the MMS position, 2 minutes after the sudden northward rotation of the IMF. The FPI plasma moments exhibit briefly the signatures of weak northward ion jets before encountering the strong southward ion jet, which was interpreted as an indication that the magnetopause X-line was close and in agreement with the predicted location from the maximum magnetic shear line.

MMS re-entered the magnetopause boundary layer at 10:09.30 UT, and a southward ion jet was observed by all spacecraft. During the MMS magnetopause boundary-layer crossing, the southward ion jet switched to a northward ion jet. This switch was first observed by the MMS1 spacecraft before it was observed by the other spacecraft, as the magnetopause X-line moved southward over the locations of the spacecraft, adjusting to the changed IMF conditions. The time delayed ion beam switch observed by the MMS spacecraft was consistent with the relative locations of the four spacecraft at the magnetopause with respect to the original location of the reconnection X-line, with MMS1 (furthest to the east) encountering the moving X-line first. The final location of the magnetopause X-line for an IMF clock angle of $316^{\circ}$ was predicted by the Maximum Magnetic Shear model to be far south of the MMS position.

As mentioned above, the Maximum Magnetic Shear model responds instantaneously to changes in the solar wind and IMF conditions, and this continuous adjustment of the X-line location at the magnetopause was confirmed for events with slow, gradual IMF rotations (Trattner et al. 2016). In contrast, the MMS data for the 19 September 2015 event also showed that during sudden rotations of the IMF, the magnetopause X-line location does not change instantaneously. In the case shown in Fig. 23, it took 6 min for the reconnection location at the magnetopause to abandon its original location and adjust to the sudden rotation of the IMF, moving southward.

A similar result was reported by Eriksson et al. (2017), who studied the response time of the dayside ionospheric convection flow after sudden IMF rotations using observations from the SuperDARN radar system. The SuperDARN observations in the auroral zone showed that the reconfiguration of the dayside ionosphere flow cells was not initiated until 11 min after the sudden IMF rotation and was not completed for another 6–8 min. While several processes were discussed to be responsible for the 17–19 min delayed response of the ionospheric convection cells (e.g., Hairston and Heelis 1995; Eriksson et al. 2017), an important factor is likely the several minutes required for the reorientation of the dayside X-line locations following a sudden IMF rotation (Trattner et al. 2016).

## The Location of Reconnection at Other Planets

Magnetic reconnection occurs at the magnetopauses of other planets. In situ observations provide evidence of reconnection at the magnetopauses of Mercury, Jupiter, Saturn, Uranus, and Neptune (Russell and Walker 1985; Huddleston et al. 1997). Anti-parallel and component reconnection have been observed at Saturn (McAndrews et al. 2008; Badman et al. 2013, Fuselier et al. 2014b, 2020), and there is evidence of transient reconnection, i.e., FTEs, at Mercury and Saturn (Imber et al. 2014; Walker and Russell 1985; Badman et al. 2013; Jasinski et al. 2016). However, there have been few attempts to systematically study the location of magnetic reconnection using the Maximum Magnetic Shear model at other planetary magnetopauses (Fuselier et al. 2014b, 2020).

**Fig. 24:**
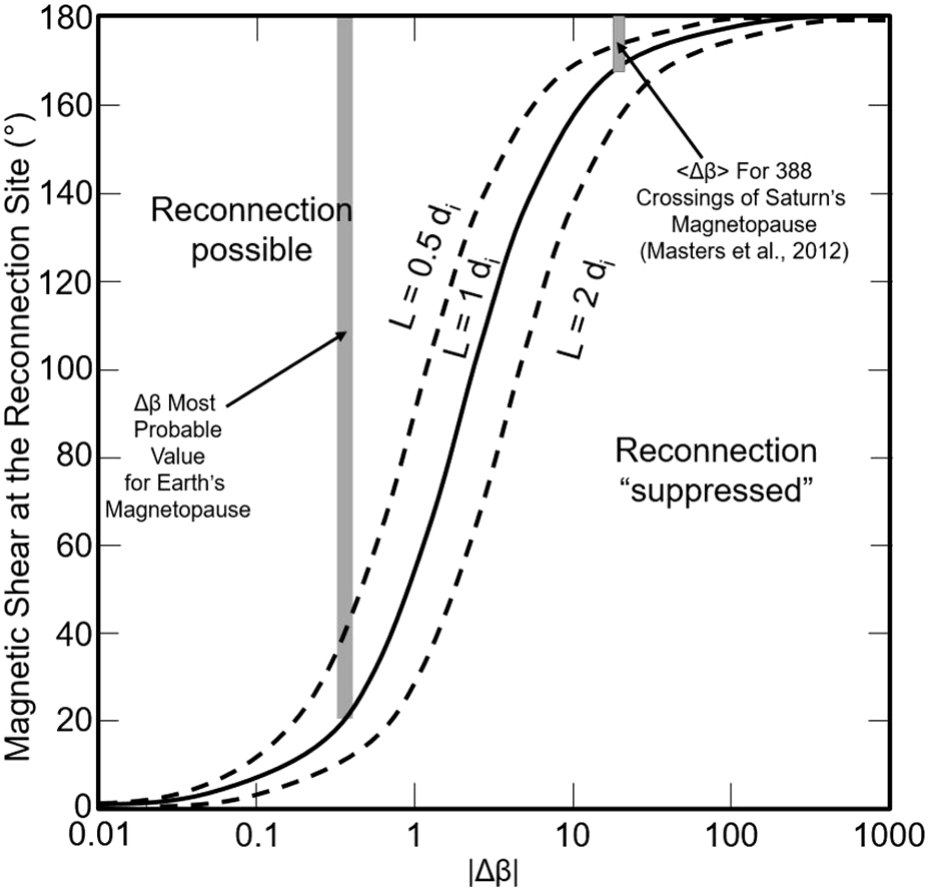
Magnetic shear angle at the reconnection site versus delta beta across the magnetopause. Magnetic reconnection is allowed in the region left of the solid or dashed “s-curves” through the middle of the plot. These three curves represent the thickness of the reconnection layer in ion inertial lengths, di (see Swisdak et al. 2003). In the region to the right of the s-curves, reconnection is suppressed. For typical solar wind conditions at Earth, reconnection is possible over a wide range of magnetic shear angles from $20^{\circ}$ to $180^{\circ}$. At Saturn, typical solar wind conditions limit reconnection to very large shear angles

One of the difficulties in direct application of the Maximum Magnetic Shear model at other magnetopauses, in particular at the magnetopauses of outer planets, is that the Maximum Magnetic Shear model requires only the IMF orientation to determine where reconnection occurs. This single vector parameter determines the reconnection location at Earth very well because plasma beta changes very little across the magnetopause from the dayside magnetosheath to the magnetosphere (Cassak and Fuselier 2016). At the outer planets, where the Mach number of the solar wind is high, plasma beta changes significantly across the magnetopause. Figure 24 shows how the change in plasma beta across the magnetopause limits the range of allowable magnetic shears for magnetopause reconnection.

In Fig. 24, the effects of diamagnetic drift of a reconnection X-line (Swisdak et al. 2003) suppress reconnection across the magnetopause for a range of magnetic shears and delta beta ($\Delta \beta $). Here $\Delta \beta $ is defined as the change in plasma beta from the magnetosheath to the magnetosphere. When the plasma and magnetic field parameters across the magnetopause are not the same, as is most often the case, reconnection at the boundary is asymmetric. Swisdak et al. (2003) proposed that this asymmetry sets conditions on the magnetic shear angle for reconnection at the magnetopause. The minimum magnetic shear angle for reconnection to occur depends on delta beta across the magnetopause. For a given change in plasma beta, reconnection is suppressed for magnetic shears that are less than this minimum shear angle.

At Earth, typical magnetosheath conditions (which are specified by typical solar wind conditions) and typical magnetospheric conditions result in relatively small $\Delta \beta $ across the magnetopause. Under typical conditions at Earth, magnetic reconnection is essentially unconstrained by plasma beta, and Fig. 24 shows that it may occur over a wide range of magnetic shear angles from $20^{\circ}$ to $180^{\circ}$ (Cassak and Fuselier 2016). Since the magnetospheric magnetic field is due north at the subsolar magnetopause, reconnection is possible for IMF clock angles from $20^{\circ}$ to $180^{\circ}$. Indeed, reconnection has been observed in the vicinity of the subsolar point for very small magnetic shear angles down to about $50^{\circ}$ (Trattner et al. 2017b).

At Saturn, solar wind and magnetosheath conditions are considerably different from those at Earth. In particular, the plasma beta in Saturn’s magnetosheath is typically much higher than that at Earth, resulting in $\Delta \beta $ values larger than 10. Figure 24 shows the average $\Delta \beta $ for 388 crossings of Saturn’s magnetopause by the Cassini spacecraft (Masters et al. 2012). The average $\Delta \beta $ results in a very narrow range of magnetic shear angles where reconnection is possible. Under these conditions, magnetic reconnection would be suppressed at the subsolar magnetopause for all but near-northward IMF (Saturn’s dipole is opposite that of Earth’s). Observations at Saturn recently demonstrated that reconnection is suppressed at the subsolar magnetopause for typical solar wind conditions (Fuselier et al. 2020).

An additional possible suppression mechanism is through plasma-flow shear at the magnetopause. At Jupiter and Saturn, magnetospheric plasma is nearly co-rotational out to the magnetopause. Thus, there may be very high flow shears at the magnetopause, particularly on the dawnside. However, a recent search for flow-shear suppression of magnetic reconnection suggested that, at times at Saturn, this type of suppression does not occur (Sawyer et al. 2019).

In conclusion, using a single parameter to determine the location of magnetic reconnection at Earth is possible ultimately because of solar wind conditions near 1 AU. At the outer planets, the possibility of magnetic reconnection forces the consideration of plasma beta at the magnetopause as a second parameter to determine where reconnection may occur at the boundary.

## Open Questions

This review paper summarizes the understanding of the magnetic reconnection location at the Earth’s dayside magnetopause. Magnetic reconnection was thought to occur in one of two different scenarios, anti-parallel (e.g., Crooker 1979) and component reconnection tilted X-line (e.g., Gonzalez and Mozer 1974). In addition to anti-parallel reconnection, more recent models predict the most likely location of the component reconnection tilted X-line by maximizing specific parameters at the magnetopause. These parameters include the reconnection outflow speed, the asymmetric reconnection outflow speed, the current density, the reconnection electric field, and the magnetic field energy in the reconnecting components (e.g., Alexeev et al. 1998; Borovsky 2013; Hesse et al. 2013; Moore et al. 2002; Schreier et al. 2010; Swisdak and Drake 2007; Teh and Sonnerup 2008).

The empirical model most widely used with observations of magnetic reconnection at the Earth’s magnetopause is the Maximum Magnetic Shear model (Trattner et al. 2007). The model combines the anti-parallel and component reconnection scenarios and predicts a long continuous X-line across the dayside magnetopause, with the section of the component reconnection tilted X-line located along the ridge of maximum magnetic shear across the dayside magnetopause.

Using confirmed observations of magnetic reconnection X-lines from the MMS mission, the Maximum Magnetic Shear model predicts the location correctly over 82% of the time. However, there are also several open questions still to be investigated.

### The Location of the X-Line Past the Terminator

In super-Alfvénic flow conditions across the Earth’s magnetopause, an X-line must move tailward so that the bulk flow speed in the plasma frame of reference where the electric fields vanish is Alfvénic. Super-Alfvénic flow conditions are usually reached past the terminator plane and there are several studies that show evidence of magnetic reconnection on the flanks of the magnetosphere (e.g., Gosling et al. 1986; Phan et al. 2000, 2006). These observations discuss accelerated plasma flow emanating from the anti-parallel reconnection region tailward of the terminator (Gosling et al. 1986) and bidirectional plasma jets at the dawn flank magnetopause which was interpreted as evidence of a stable reconnection line (Phan et al. 2000, 2006).

A study by Gomez et al. (2016) discussed a stable X-line location observed by the MMS spacecraft at the dusk terminator with an ambient plasma flow that slightly exceeds the local Alfvén speed. Observed gradual changes of the magnetopause X-line location were the result of a slow rotation of the IMF clock angle, which caused the X-line to move against the super-Alfvénic magnetosheath bulk flow direction.

When the IMF is strongly northward ($310^{\circ} < \text{IMF CA} < 50^{\circ}$), steady magnetic reconnection does not occur equatorward of the cusps. Rather, it occurs tailward of the cusps, as first demonstrated with Hawkeye observations by Kessel et al. (1996). The strongly northward IMF condition causes the draped magnetosheath magnetic field to increase and the plasma density to decrease near the magnetopause. This region is known as a magnetic barrier (Spreiter and Alksne 1969; Erkaev 1988) or a plasma depletion layer (Zwan and Wolf 1976), and reduces the local Alfvén speed just outside the magnetopause such that sub-Alfvénic flow can exist tailward of the cusps (Fuselier et al. 2000; Petrinec et al. 2003; Lavraud et al. 2005). Observational studies have also shown that high-latitude magnetic reconnection occurs where the magnetosheath and magnetospheric magnetic field lines are nearly anti-parallel (e.g., Gosling et al. 1991; Fuselier et al. 2002; Phan et al. 2003; Bobra et al. 2004; Trattner et al. 2004).

Using global MHD simulations, Siscoe et al. (2002) discusses a possible mode for magnetic reconnection dubbed flow-through-reconnection (FTR). In the simulation environment, the magnetic field effectively disengages from the plasma flow allowing the plasma to stream through the reconnection region at super-Alfvénic speeds.

While X-lines in a super-Alfvénic flow regime are expected to move with the flow, mitigating circumstances, e.g. the formation of plasma depletion layers during northward IMF conditions, have been shown to allow for stable X-line locations. During southward IMF conditions, an X-line located in the super-Alfvénic regime at the flanks of the magnetopause runs along the anti-parallel reconnection region. However, the orientation of the anti-parallel reconnection region is parallel to the super-Alfvénic magnetosheath plasma flow direction, which might result in very different X-lines compared to X-lines at the dayside magnetopause.

The extent of anti-parallel reconnection along the magnetospheric flanks and the stationarity of reconnection X-lines in the presence of super-Alfvénic flow are questions that result from a general lack of understanding of reconnection in the presence of shear flow.

### The Orientation of the X-Line

**Fig. 25:**
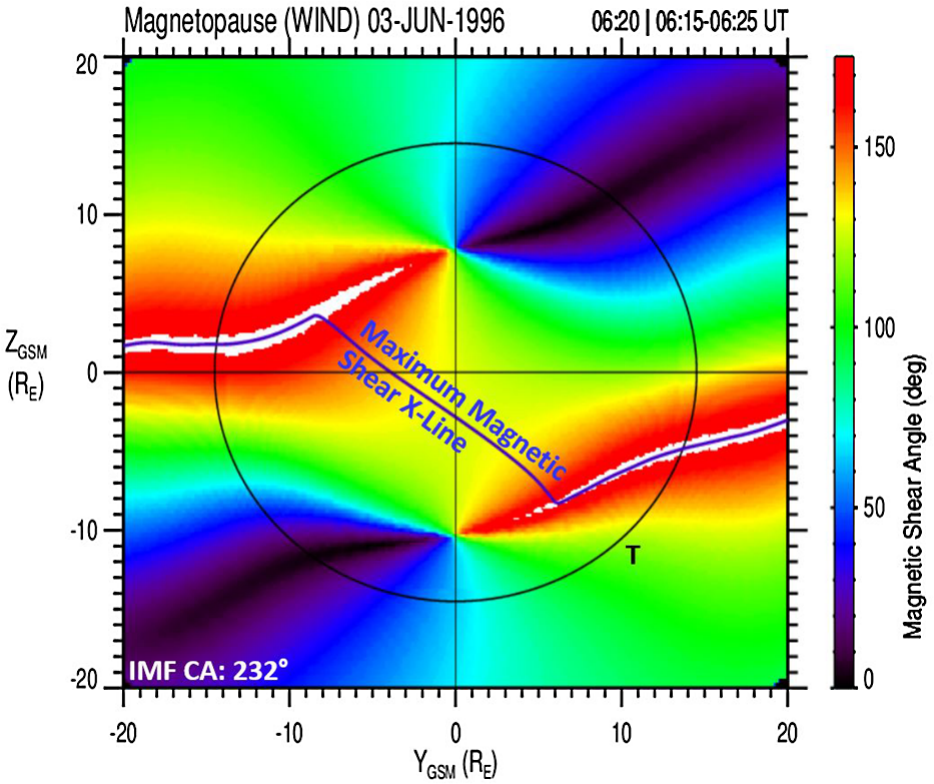
Magnetopause magnetic shear angle plot for 3 July 1996 at 06:20 UT. The blue line represents the location of the maximum magnetic shear X-line, combining the anti-parallel reconnection location at the flanks with a component reconnection tilted X-line crossing the dayside magnetopause

In the classical concept of magnetic reconnection as depicted in Figs. 1 and 6, the orientation of the X-line is thought to be perpendicular to the “ideal” anti-parallel magnetic field configuration. However, some magnetopause magnetic shear conditions as the one shown in Fig. 16, the “Knee” events, make such a scenario impossible. During a “Knee” event, the magnetopause anti-parallel reconnection branches do not run directly from the equator at the terminator plane to one of the magnetospheric cusps, but bends with variable slopes towards the cusps in such a manner that an anti-parallel reconnection branch can align along the draped IMF field lines. Thus, while the “reconnection X-line” is drawn as a continuous line stretching from the dawn to dusk flanks (see Fig. 25), there are parts of this X-line that may not be continuous and parts that may not be oriented in the direction shown.

### The Location of the X-Lines During Fast Southward Rotations of the IMF

The reconnection scenarios at the Earth’s magnetopause during dominant IMF B_Y_ and IMF B_Z_ conditions are predicted (and observed) to be the component reconnection tilted X-line and the anti-parallel reconnection X-line, respectively. It is currently not understood how magnetic reconnection at the Earth’s magnetopause transitions between the two scenarios during fast or even slow southward rotations of the IMF where the B_Z_ component rapidly or slowly becomes more negative and dominates. Magnetopause reconnection could either continuously morph from the component reconnection scenario into the anti-parallel reconnection scenario or the component reconnection scenario may be abandoned until new anti-parallel reconnection locations are established after the rotation of the IMF is completed. Similarly, the rotation from B_Z_ dominant conditions to B_Y_ dominant conditions should produce a component reconnection line across the noon meridian. However, it is not clear if this reconnection line forms rapidly across a wide range of local times or grows more slowly away from the anti-parallel reconnection regions.

### Reconnection Location Anomalies for Specific IMF Clock Angles During Equinox

For magnetopause reconnection events observed around the equinoxes (no dipole tilt), the Maximum Magnetic Shear model predicts that the component reconnection tilted X-line should cross the dayside magnetopause in the vicinity or at the subsolar point location. Several large surveys (Trattner et al. 2007, 2017a) did show a subsolar location of the component reconnection tilted X-line but also revealed a persistent anomaly within the model where the observed X-line is far away from the subsolar point. This anomaly only occurs for events with IMF clock angles around $120^{\circ}$ and $240^{\circ}$, during the spring and fall equinoxes, respectively, and has not been solved.

### Magnetic Reconnection at the Earth’s Magnetopause During Large $\Delta \beta $ Across the Magnetopause

Figure 24 shows the parameter space where reconnection is allowed as a function of the local magnetic shear angle and the plasma beta across the magnetopause boundary layer $\Delta \beta $. The Figure also shows the most probable $\Delta \beta $ values encountered at Earth and Saturn. From these most probable values, it is evident why reconnection at the Earth’s magnetopause is seemingly always present and can even occur for very low magnetic shear angles. For high $\Delta \beta $ conditions at the Earth’s magnetopause, it is not known if suppression of the component reconnection tilted X-line occurs like at Saturn (Fuselier et al. 2020).

### Magnetic Reconnection During Dominant IMF B_X_ Conditions

Predicting the reconnection location for dominant IMF B_X_ conditions ($|\text{B}_{\text{X}}|/\text{B} > 0.7$) has thus far proven to be elusive. In the original study that led to the development of the Maximum Magnetic Shear model (Trattner et al. 2007), the limited number of dominant IMF B_X_ events seem to have reconnection sites in the high-latitude, anti-parallel reconnection region close to the cusp. With more events being analyzed and new data from MMS, the predictions of the reconnection locations have had mixed success, with some events occurring in the anti-parallel region and others in the component region spanning the dayside magnetopause. The mixed results led to the conclusion that additional parameters might play a role in defining the reconnection location for dominant IMF B_X_ conditions.

## Data Availability

Solar wind observations were provided by the Wind “Solar Wind Experiment” (Wind/SWE) (Ogilvie et al. 1995). The IMF measurements were provided by the Wind “Magnetic Field Instrument” (Wind/MFI) (Lepping et al. 1995). The solar wind data are available at CDAWeb (http://cdaweb.gsfc.nasa.gov/istp_public/). All MMS data presented in this study are available to the general public through the MMS data website (https://lasp.colorado.edu/mms/sdc/public/about/).
